# The poleward enhanced Arctic Ocean cooling machine in a warming climate

**DOI:** 10.1038/s41467-021-23321-7

**Published:** 2021-05-20

**Authors:** Qi Shu, Qiang Wang, Zhenya Song, Fangli Qiao

**Affiliations:** 1 First Institute of Oceanography, and Key Laboratory of Marine Science and Numerical Modeling, Ministry of Natural Resources, Qingdao, China; 2 Laboratory for Regional Oceanography and Numerical Modeling, Pilot National Laboratory for Marine Science and Technology, Qingdao, China; 3 Shandong Key Laboratory of Marine Science and Numerical Modeling, Qingdao, China; 4 Alfred Wegener Institute Helmholtz Centre for Polar and Marine Research (AWI), Bremerhaven, Germany

**Keywords:** Cryospheric science, Physical oceanography

## Abstract

As a cooling machine of the Arctic Ocean, the Barents Sea releases most of the incoming ocean heat originating from the North Atlantic. The related air-sea heat exchange plays a crucial role in both regulating the climate and determining the deep circulation in the Arctic Ocean and beyond. It was reported that the cooling efficiency of this cooling machine has decreased significantly. In this study, we find that the overall cooling efficiency did not really drop: When the cooling efficiency decreased in the southern Barents Sea, it increased in the northern Barents and Kara Seas, indicating that the cooling machine has expanded poleward. According to climate model projections, it is very likely that the cooling machine will continue to expand to the Kara Sea and then to the Arctic Basin in a warming climate. As a result, the Arctic Atlantification will be enhanced and pushed poleward in the future.

## Abstract (web summary)

The Arctic Ocean cooling machine, currently the Barents Sea, plays a crucial role in both regulating the climate and determining the deep ocean circulation. Here the authors show that the efficiency of the cooling machine is poleward enhanced in a warming climate, which pushes Arctic Atlantification poleward.

## Introduction

The Arctic Ocean is located at the northern end of the Global Conveyor Belt. The North Atlantic provides the cold Arctic Ocean with ocean heat in two Atlantic Water (AW) branches^1^. One branch passes through the Fram Strait and supplies the warm AW layer of the Arctic Ocean^2–4^. The other enters the Barents and then Kara Seas, and finally flows to the intermediate and deeper layers of the Arctic Ocean^5–7^. Unlike the Fram Strait branch, almost all the AW heat in the Barents Sea branch is already released to the atmosphere inside the Barents Sea, which functions as a big cooling machine for the Arctic Ocean^8,9^. The released ocean heat over the Barents Sea could significantly influence the climate and occurrence of extreme events over the Northern Hemisphere midlatitude continents, and the deep circulation in the Arctic Ocean and beyond^8,10–15^. Recently, it was reported that the cooling efficiency in the Barents Sea has decreased over the past decades, with potential impacts on the ocean circulation in the adjacent ocean basins^16^.

A phenomenon called Arctic Atlantification has been witnessed in the Eurasian sector of the Arctic Ocean recently^17–19^. It is characterized by significant ocean warming and weakening in upper ocean stratification along with winter sea ice decline. The Arctic Atlantification has been causing a shrinking of the Arctic marine biome and rapid borealization of fish communities in the Arctic Ocean^20–22^. The weakened stratification associated with the Arctic Atlantification can facilitate the upward release of ocean heat and enhance Arctic sea ice decline^18,23^, which can further amplify Arctic warming^24–27^. The surface  ocean in some of the Arctic areas will become fresher in the future due to an enhanced water cycle in the Earth System^28–30^, which can stabilize the upper ocean. The future evolution of the upper ocean stratification, one of the most important factors determing marine primary productivity^31^ and dense water formation, which drives large-scale ocean circulation^32,33^, depends on the relative strength of the opposite effects of Arctic Atlantification and surface freshening. Therefore, how the Arctic Atlantification will proceed in the future is of great relevance to the Northern Hemisphere climate and marine ecosystem.

A sole reduction in surface cooling efficiency means a more stable ocean, a tendency opposite to that in the progress of Arctic Atlantification. In this study, by analyzing an ocean reanalysis dataset and historical and Shared Socioeconomic Pathway 585 (SSP585) scenario simulations of the Coupled Model Intercomparison Project Phase 6 (CMIP6), we find that the overall cooling efficiency of the Arctic Ocean cooling machine did not decrease. Instead, the cooling machine has just expanded poleward with an increase in its overall cooling efficiency, and will likely continue to expand further north in a future warming climate. This expansion can cause more ocean heat to be released at higher latitudes, where sea ice cover will be reduced and the upper ocean stratification will be weakened in wintertime, manifesting a strengthened poleward advance of the Arctic Atlantification.

## Results

### Arctic Ocean cooling machine in the past

**Fig. 1:**
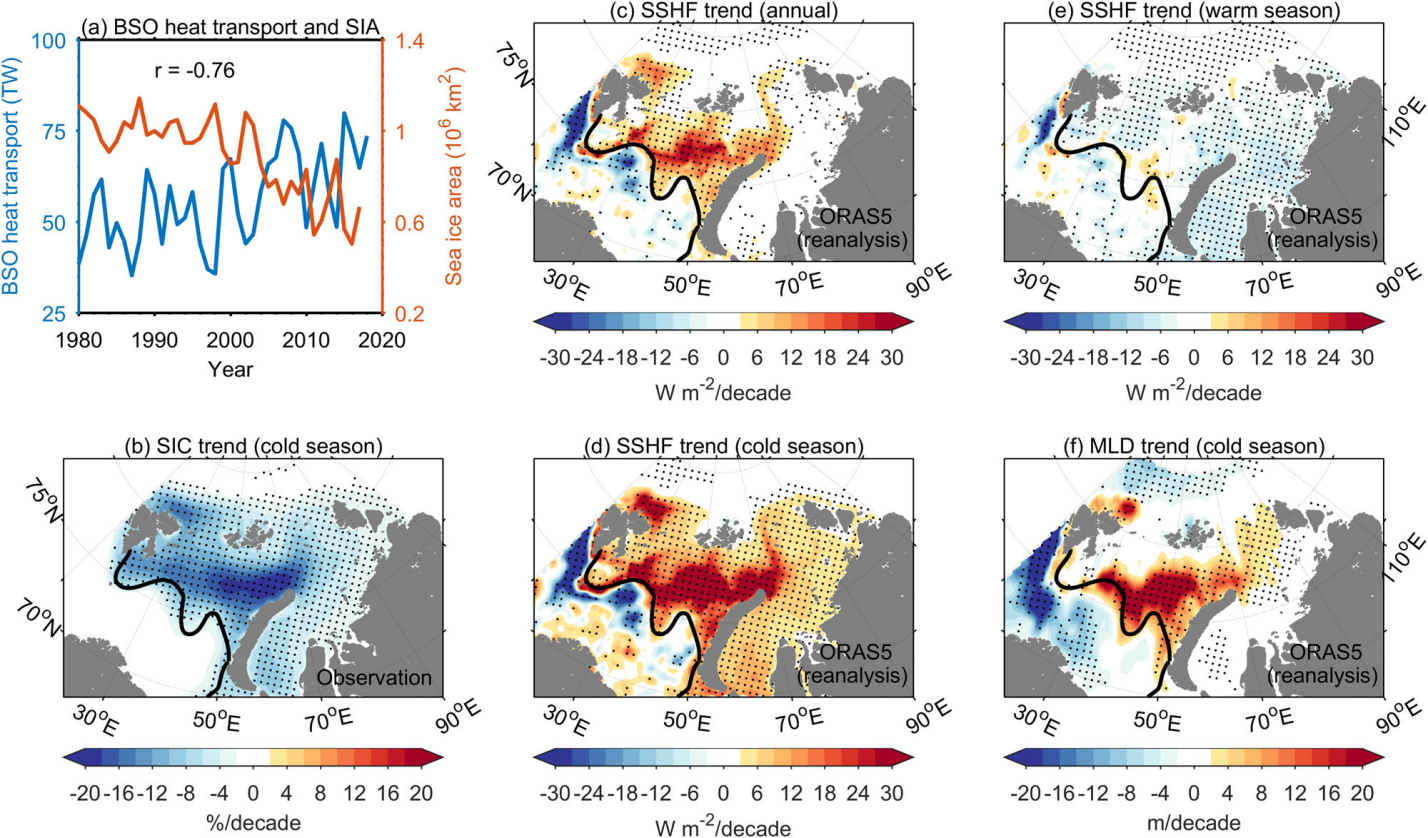
Climate change in the Barents and Kara Seas in the past. **a** The time series of ocean heat transport through the Barents Sea Opening (BSO) and the cold season (October–March) sea ice area (SIA) in the Barents and Kara Seas. The linear trends of **b** cold season sea ice concentration (SIC), **c** annual mean sea surface heat flux (SSHF), **d** cold season sea surface heat flux, **e** warm season (April–September) sea surface heat flux, and **f** cold season mixed layer depth (MLD) of 1979/80–2017/18. Dots indicate that linear trends exceed the 95% confidence level. The black lines in **b**–**f** are the 0 °C surface isotherm based on climatology from WOA13, which can represent the boundary between the southwestern and northern Barents Sea^14^. Upward sea surface heat flux is positive. Ocean heat transport (**a**), SSHF (**c**, **e**, **d**), and MLD (**f**) are from the Ocean ReAnalysis System 5 (ORAS5). Satellite observed SIA (**a**) and SIC (**b**) are from the National Snow and Ice Data Center.

The trend of AW volume transport into the Barents Sea was not significant over the past decades, but the strong warming trend in the inflow (Supplementary Fig. 1) led to a significant upward trend in the heat transport^16,34^. More inflowing ocean heat can result in less sea ice formation in the cold season in the Barents and Kara Seas^17,35^, thus leading to a high anticorrelation between AW heat inflow and cold season sea ice area (*r* = −0.76, *p* value is <0.01, see Fig. 1a, b). This explains the success in winter sea ice prediction, using poleward AW heat transport^36,37^. Consistent with the warming trend in the AW inflow, the southern Barents Sea has become warmer over the past few decades (Supplementary Fig. 1).

In the southwestern Barents Sea, annual mean ocean surface heat flux has negative trends over the past four decades (Fig. 1c), which originate from the trends in the cold season (Fig. 1d, e) and indicate a reduction in the efficiency of the cooling machine in this region^16^. Here, we take the convention that upward heat flux (sea surface heat loss) is positive. The negative trends in surface heat loss in the cold season are mainly because the near-surface air has a stronger warming trend than the ocean (Supplementary Fig. 2a). The strong air warming trend can be attributed to both local feedbacks and increasing atmosphere heat and moisture transport from lower latitudes^38–40^. As a consequence of weakened ocean surface cooling, the upper ocean in the southwestern Barents Sea became more stable as depicted by the decrease in surface mixed layer depth (MLD, Fig. 1f).

**Fig. 2:**
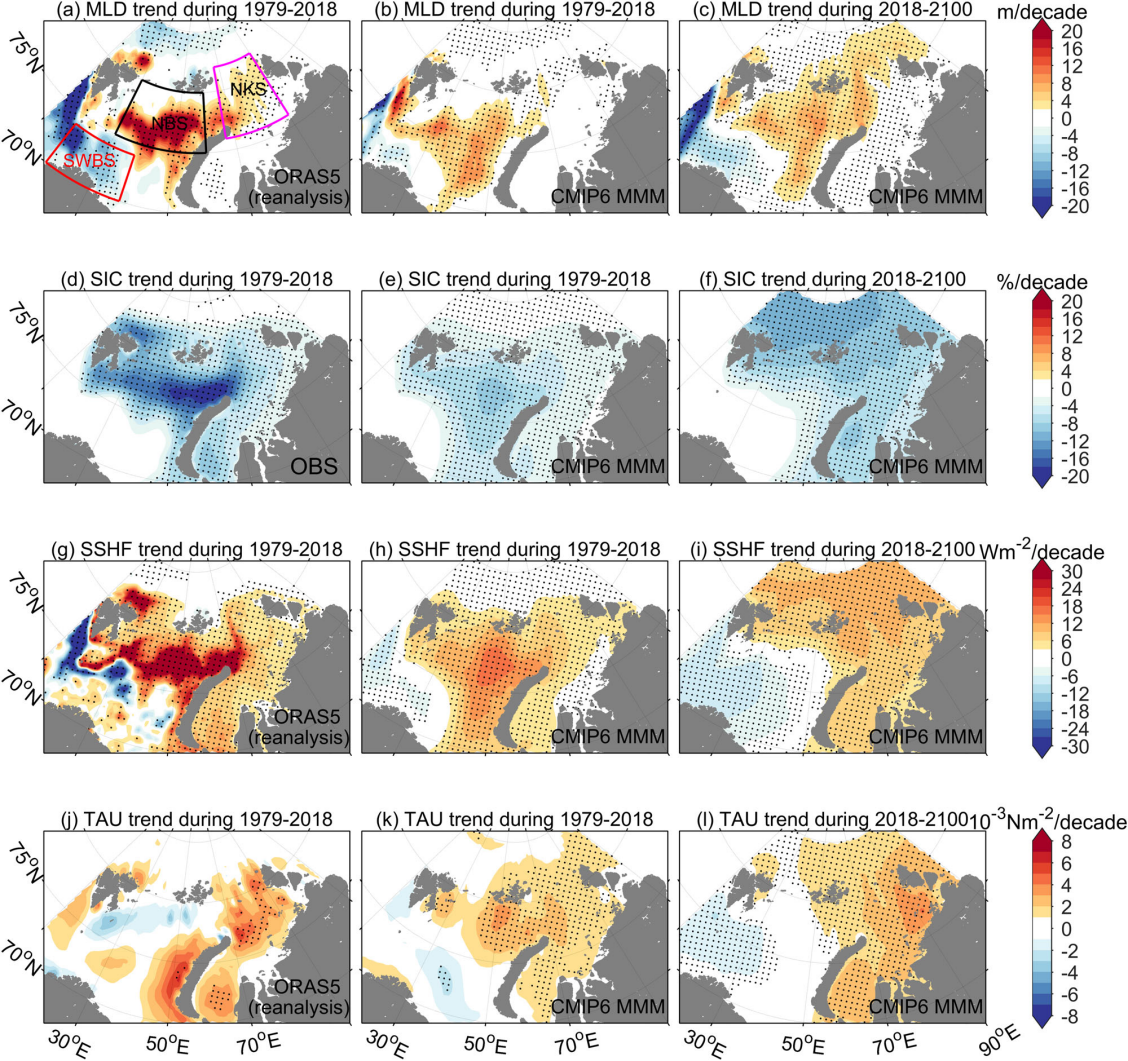
Climate change in the CMIP6 models compared with observation and reanalysis. **a**–**c** The linear trend of cold season mixed layer depth (MLD), **d**–**f** sea ice concentration (SIC), **g**–**i** sea surface heat flux (SSHF), and **j**–**l** sea surface wind stress (TAU) in the observation (OBS), reanalysis and CMIP6 multi-model mean (MMM). Dots indicate that linear trends exceed the 95% confidence level. Upward sea surface heat flux is positive. The red, black, and magenta boxes in **a** represent the southwestern Barents Sea (SWBS, that is, the Barents Sea Opening area), northern Barents Sea (NBS), and northern Kara Sea (NKS), respectively, which are used in Figs. 3 and 4, and Supplementary Figs. 5 and 6.

Opposite to the changes in the southwestern Barents Sea, annual mean ocean surface heat flux along the AW pathway in the northern Barents and Kara Seas has pronounced upward trends over the past four decades (Fig. 1c), due to enhanced heat loss in the cold season and relatively small trends in the warm season (Fig. 1d, e). Lower sea ice concentration results in more open water exposed to the cold air above (Fig. 1b), so ocean heat is released more efficiently (Fig. 1d), mainly through sensible and latent heat fluxes. Figure 1d depicts that over the past 40 years there is an increase in the heat loss in the northern Barents and Kara Seas, and the area of effective cooling thus appears to expand northward. More surface heat loss in winter can cause stronger convection, thus a deeper mixed layer in the northern Barents and Kara Seas (Fig. 1f). The colocation of strong decreasing trends in sea ice concentration, and increasing trends in the MLD in the northern Barents and Kara Seas (Fig. 1b, f) well demonstrates the occurrence of Arctic Atlantification in these regions. Ocean surface stress has positive trends in some areas of the Barents and Kara Seas (Fig. 2j). Although vertical mixing induced by ocean surface stress can also contribute to stronger convection and deeper mixed layer, the low spatial correlation between the trends of surface stress and MLD in the reanalysis indicates that surface stress plays a less important role than sea ice decline and surface cooling. Here, we identified a weakening of stratification in wintertime along the AW pathway in both the northern Barents and Kara Seas. In late summer and early autumn, the stratification in the northern Barents Sea has also weakened, which is believed to be caused by a reduction in sea ice meltwater associated with the decline in sea ice volume import^41^.

**Fig. 3:**
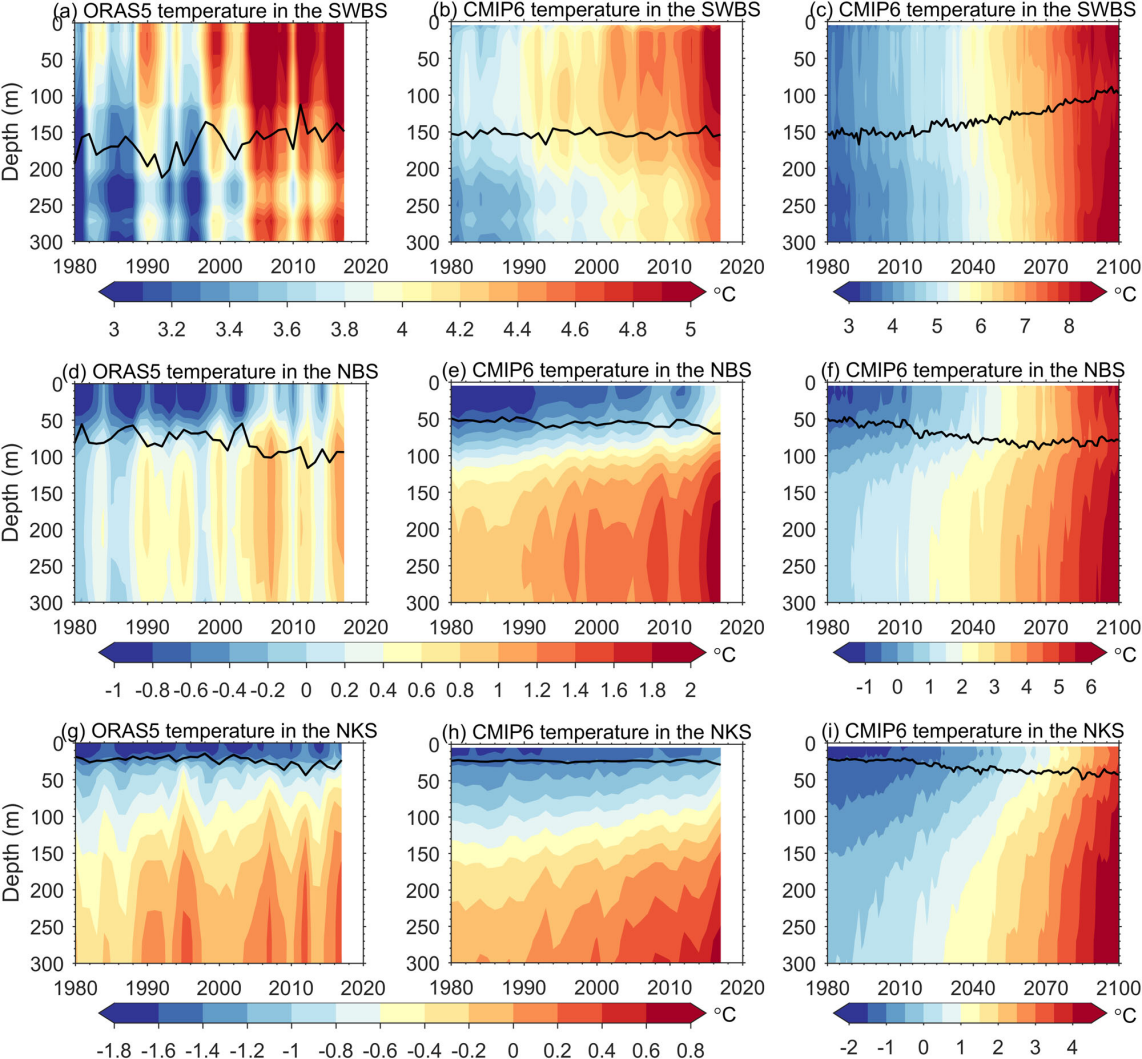
Temperature changes in the southwestern Barents Sea, northern Barents Sea, and northern Kara Sea. **a**–**c** Depth-time plots of cold season temperature averaged in the southwestern Barents Sea (SWBS, the red box shown in Fig. 2a) in the reanalysis (ORAS5) and CMIP6 MMM. **d**–**f** The same as **a**–**c**, but for the northern Barents Sea (NBS, the black box shown in Fig. 2a). **g**–**i** The same as **a**–**c**, but for the northern Kara Sea (NKS, the magenta box shown in Fig. 2a). The black lines show the mixed layer depth (MLD) averaged in the respective boxes.

Both the increase in AW heat inflow and reduction in ocean surface cooling can lead to ocean warming in the Barents and Kara seas^42^. Over the past four decades, the increased heat gain in the southwestern Barents Sea through AW inflow was not compensated by surface heat loss, which had a negative trend (Fig. 1c), resulting in a strong ocean warming trend (Fig. 3a). Although the AW lost more heat by melting more sea ice and releasing more heat to the atmosphere along its pathway further to the north (Fig. 1b, c), the ocean also became warmer in all the depth ranges in both the northern Barents and Kara Seas (Fig. 3d, g). Some of the AW in the Fram Strait branch can enter the northern shelf of the Barents Sea^43,44^ and the St. Anna Trough^45^ from the north. An upward trend in ocean temperature and heat transport has been found upstream in the West Spitsbergen Current^2,46^, so this branch may also contribute to the warming and increase of ocean heat release in the northern Barents and Kara Seas. However, the strongest increasing trends in ocean surface heat loss and surface MLD are along the pathway of the Barents Sea branch AW, which extends from the inner shelf of the northern Barents Sea to the northern Kara Sea (Fig. 1d, f). Therefore, these trends are mainly associated with the Barents Sea branch AW.

The consistent changes in winter MLD, sea ice concentration, surface heat flux, ocean temperature, and heat content (Fig. 2b, e, h, and Supplementary Figs. 3 and 4) in the Barents and Kara Seas over the past four decades are reproduced by the multi-model mean (MMM) of CMIP6 coupled models. This indicates that the Arctic Atlantification over the past four decades represents a signal of forced climate change. However, both the negative trends in the southwestern Barents Sea and positive trends in the northern Barents and Kara Seas for ocean surface heat flux and MLD are weaker in the CMIP6 MMM than in the reanalysis. Meanwhile, the sea ice declining trend is also underestimated in the CMIP6 models. The weaker trends in the MMM can be due to that internal climate variability is responsible for part of the observed sea ice decline, and that climate models tend to underestimate the AW heat transport into the Barents Sea^47^.

### Arctic Ocean cooling machine in the future

CMIP6 projections under the SSP585 scenario clearly indicate that the AW warming (Supplementary Figs. 3 and 4) and the Arctic Atlantification will continue in the 21st century (Fig. 2c, f, i). Over the past 40 years, the most rapid sea ice decline and the fastest increase in ocean heat release mainly occurred in the northern Barents Sea (Fig. 2d, e, g, h). In the future, the upward trends in winter surface heat loss and decreasing trends in winter sea ice cover will continue to proceed poleward (Fig. 2c, f, i).

The ocean salinity in the Barents and Kara Seas has a significant decreasing trend in the future based on the CMIP6 MMM, which is the most pronounced in the upper ocean (Supplementary Fig. 5c, f, i). The Arctic freshening trend can be attributed to the increase in Arctic freshwater sources, including sea ice meltwater, river runoff, precipitation, and freshwater transport through Barents Sea Opening^30^. As a consequence, the potential density in the Barents and Kara Seas will decrease in the future (Supplementary Fig. 6). Despite the stabilization of the ocean by the freshening trend, the winter MLD along the AW pathway in the northern Barents and Kara Seas will increase in the 21st century (Figs. 2c and 3f, i). This reveals that the destabilization induced by enhanced heat loss associated with the reduced sea ice cover is strong enough to overwhelm the stabilization by surface freshening. In the southwestern Barents Sea, as the haline stratification increases and surface cooling weakens in the future, the winter MLD in this region will continue to decrease (Figs. 2c and 3c). Ocean surface stress in the Barents and Kara Seas will increase in the future as shown by the CMIP6 MMM (Fig. 2l). Thinner and less compact sea ice could enhance air–sea momentum transfer and increase ocean surface stress^48,49^. However, the strongest increase in ocean surface stress is not in the region where winter MLD has the largest trend, implying that it will play a less important role in changing the upper ocean stratification than surface cooling in the future.

**Fig. 4:**
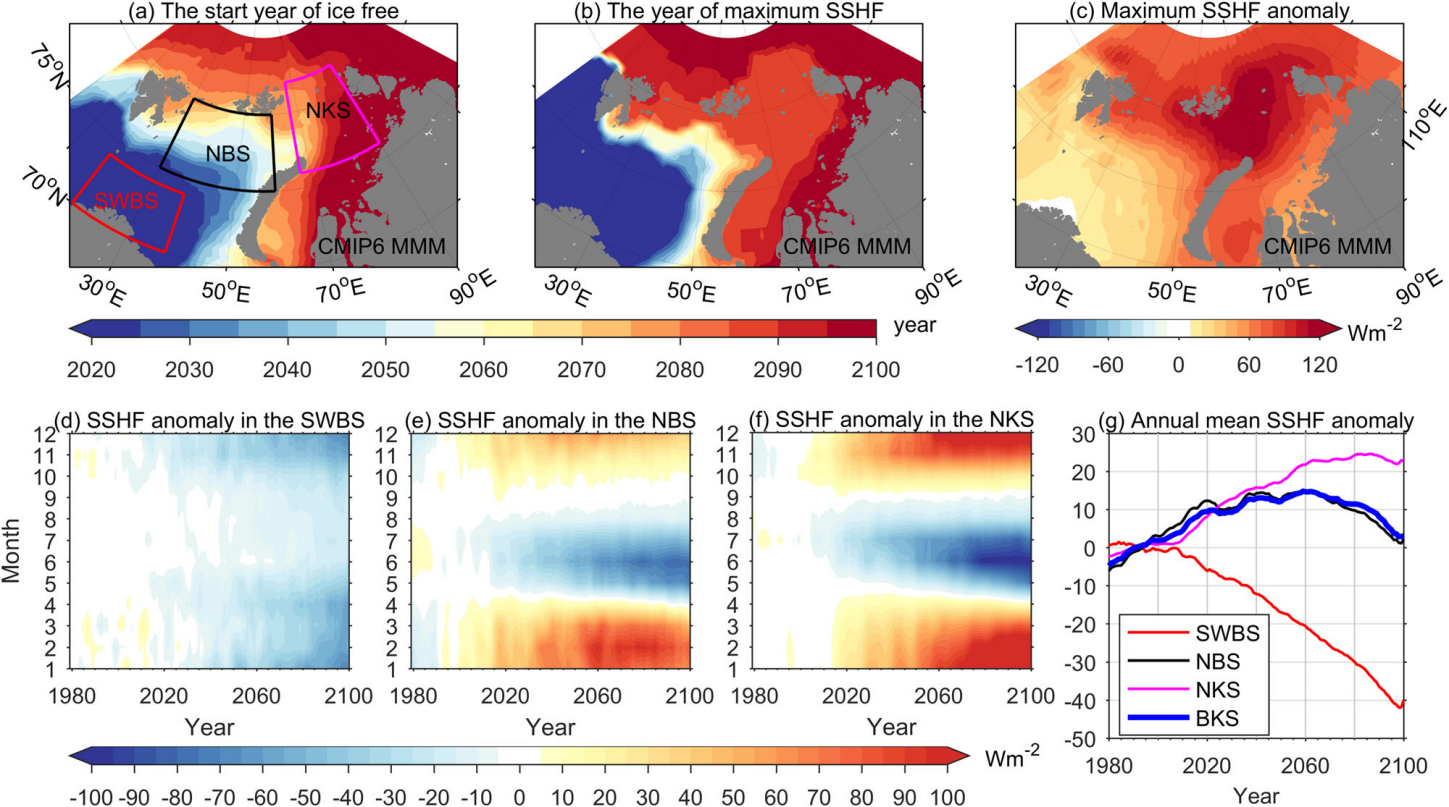
The temporal and spatial evolution of the Arctic Ocean cooling machine. **a** The start year of being ice-free in the cold season during 2018/19–2099/2100. **b** The year of maximum cold-season sea surface heat flux (SSHF) anomalies during 2018/19–2099/2100. **c** The maximum of cold-season sea surface heat flux anomalies during 2018/19–2099/2100 referenced to the climatology. **d**–**f** Thirteen-year smoothed monthly sea surface heat flux anomalies averaged in the southwestern Barents Sea (SWBS), northern Barents and Kara Seas (NBS and NKS). **g** Thirteen-year smoothed annual mean sea surface heat flux anomalies averaged in the SWBS, NBS, NKS, and the entire Barents and Kara Seas (BKS). Anomalies are referenced to the mean over the period 1979–2008. The red, black, and magenta boxes in **a** indicate the areas used in **d**, **e**, and **f**, respectively.

Under the SSP585 scenario, the northern Barents Sea will be ice-free in winter in ~2060, while the Kara Sea will become ice-free in winter in the 2080s except for the southeastern Kara Sea (Fig. 4a), which is not directly influenced by the warm AW. In the past decades, the southwestern Barents Sea has been losing efficiency in ocean cooling, and efficient ocean surface heat loss has been expanding to the northern Barents Sea (Fig. 1c, d). The cooling efficiency in the southwestern Barents Sea will continue decreasing in a future warming world (Fig. 4d, g), while the cooling efficiency in the northern Barents Sea will continue increasing for some decades and will eventually start to drop after reaching the maximum cooling efficiency in ~2060 (Fig. 4b, e, g). Efficient surface cooling will continue to expand into the northern Kara Sea along with the decline of winter sea ice. The northern Kara Sea will have the strongest increase in winter surface heat loss compared with the areas to the southwest (Fig. 4c–f). The cooling efficiency in the northern Kara Sea will reach its maximum after this region is ice-free in winter in the 2080s (Fig. 4a, b, f, g). Averaged over the entire Barents (area bounded by 18–60° E and 66–81° N) and Kara (area bounded by 60–100° E and 66–84° N) Seas, the overall cooling efficiency will continue increasing until ~2060 and begin to drop significantly starting from the 2080s (Fig. 4g). The latter indicates that the Arctic Ocean cooling machine might expand to the Arctic Basin in the 2080s.

Consistent with the changes in the magnitude of winter surface heat loss, the winter MLD in the northern Barents Sea will increase until about 2060s (Fig. 3f), while it will continue to increase until the 2080s in the northern Kara Sea in the SSP585 scenario (Fig. 3i). That is, the temporal evolution of the MLD, an important indicator of the poleward expansion of the Arctic Atlantification, is synchronized with that of the Arctic Ocean cooling machine. However, the magnitude of the MLD will remain the largest in the southwestern Barents Sea and smallest in the northern Kara Sea within the 21st century, due to spatially decreasing surface salinity from the southwestern Barents Sea toward the Arctic Basin (Supplementary Fig. 5).

**Fig. 5:**
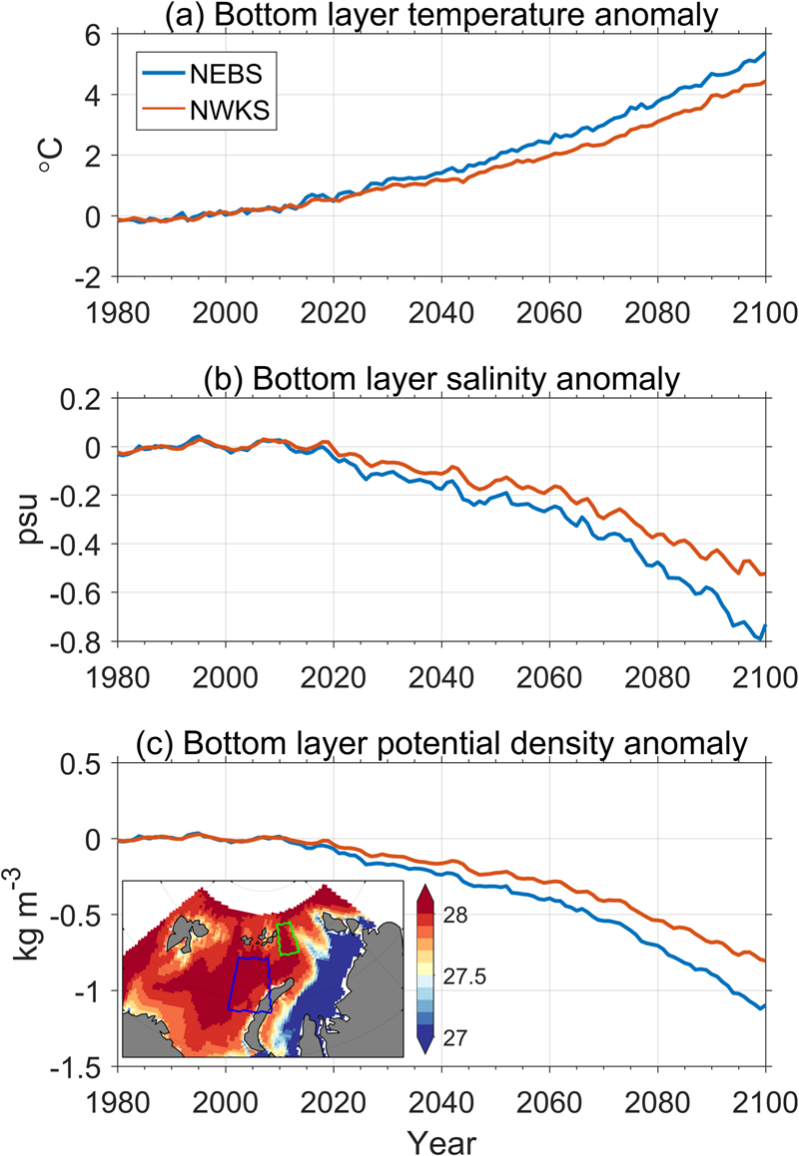
Climate change of Barents Sea Water. Anomalies of CMIP6 MMM bottom layer temperature (**a**), salinity (**b**), and potential density referenced to surface (**c**) in the northeastern Barents Sea (NEBS) and northwestern Kara Sea (NWKS). Anomalies are referenced to the mean over the period 1979–2008. The insert panel in **c** depicts CMIP6 MMM climotology of bottom layer potential density averaged over the period of 1979–2018. The blue and green boxes in the insert panel in **c** represent the NEBS and NWKS, respectively.

The northern Barents and Kara Seas have a warming trend in all the depth ranges through the 21st century (Fig. 3f, i), despite the changes in ocean surface heat loss. Barents Sea Water (BSW), the dense water formed in the Barents Sea that feeds the Arctic intermediate layer and the large-scale ocean circulation^8,14,15^, will also continue to warm up in the future warming climate as indicated by the change in the MMM ocean bottom temperature in the northeastern Barents Sea (NEBS, Fig. 5a). At the end of the 21st century, the MMM bottom temperature in the NEBS predicted in the SSP585 scenario will be >5 °C warmer than the climatology. The freshening trend in the water column (Supplementary Fig. 6) also reaches the ocean bottom layer (Fig. 5b). The warming and freshening trends together cause a strong declining trend in BSW density (Fig. 5c). In the northwestern Kara Sea, the future changes of the BSW are similar to those in the NEBS, although the amplitudes are smaller (Fig. 5). With the strong warming trend, the BSW will become an important heat source for the Arctic Basin. The reduction in BSW density also implies a changing impact on Arctic intermediate water and large-scale ocean circulation.

**Fig. 6:**
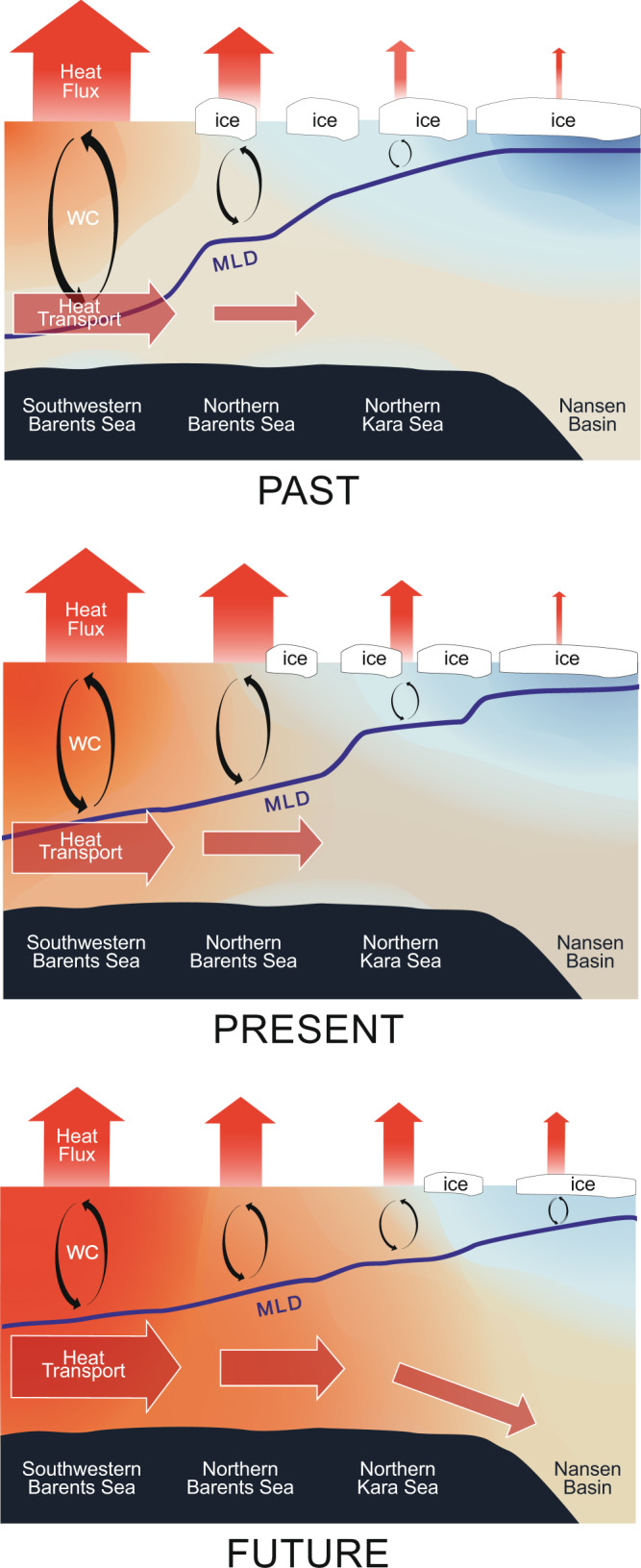
Poleward enhanced cooling machine and Arctic Atlantification. Conceptual schematic showing the related processes: ocean heat transport from the upstream increases because of the warming of the Atlantic Water inflow, but ocean surface heat release in the ice-free regions decreases due to more rapid atmosphere warming. Consequently, the mixed layer depth associated with winter convection decreases in the ice-free regions, and more heat is left in the ocean, leading to enhanced ocean warming in the downstream region. The latter induces stronger sea ice decline, more intensive ocean surface heat loss, and deeper mixed layer depth in winter in the downstream region. WC and MLD stand for winter convection and mixed layer depth, respectively.

The future evolution of the Arctic Ocean cooling machine and its impact on the Arctic Atlantification in a warming climate can be summarized as follows (Fig. 6). Despite the warming trend of the AW inflow, the surface cooling efficiency will decrease in the upstream region of the AW pathway due to atmosphere warming (Supplementary Fig. 2c). Then more heat will be reserved in the ocean to enhance the ocean warming in the downstream region. This will strengthen winter sea ice decline, thus leading to larger open ocean areas and stronger ocean surface cooling in winter in the downstream region, representing a poleward expansion of the Arctic Ocean cooling machine. Therefore, the reduction in the cooling efficiency in the upstream region, which has started already^16^ and will continue in the future, preconditions the increase in the cooling efficiency in the downstream region and is part of the process of the poleward expansion of the cooling machine. The resultant enhancement in both the ocean warming and MLD increase in the downstream region concurrently enhances the poleward advance of the Arctic Atlantification. The enhanced warming in the ocean reaches the BSW at the bottom, with potential impacts on the Arctic Basin at depth.

## Discussion

Most of the CMIP6 models consistently show a poleward advance of the Arctic Ocean cooling machine and Arctic Atlantification (Supplementary Figs. 7–14). The significant model spreads in the simulated linear trends of sea ice concentration, sea surface heat flux, MLD, and sea surface stress (Supplementary Fig. 15) imply possible uncertainties in the predicted timing and strength of the changes in the cooling machine and Arctic Atlantification represented by the MMM. In particular, the underestimated trends in sea ice decline, ocean surface heat flux, and MLD in the CMIP6 MMM compared to observations and reanalysis as shown in Fig. 2 imply that the future development of the poleward expansion of the cooling machine and the strengthening of Arctic Atlantification are very possibly underestimated in the CMIP6 models on average. The low resolution used in climate models can lead to underestimation of ocean heat transport to the Barents Sea, which was suggested to be one of the main reasons for the too weak decreasing trend in sea ice cover in model simulations^47,50^. The future development of upper-ocean stratification is determined by both the changes in surface heat loss and surface salinity. The CMIP6 models have a large spread in the simulated sea surface salinity, for example, in the southwestern Barents Sea (Supplementary Fig. 16). Salinity in the AW transported to the Barents Sea is associated with the strength of Atlantic Meridional Overturning Circulation, which has a large model spread in CMIP6 simulations^51^. Furthermore, salinity in the northern North Atlantic and the Arctic Ocean is also significantly influenced by the poleward atmospheric moisture transport^52,53^. Therefore, the model spread in the surface salinity in the Barents Sea could have origins in both the large-scale ocean and atmosphere circulations in the models. Using higher model resolution and improved physical parameterizations in different components of climate models are required to reduce projection uncertainties in the next phase of CMIP.

AW also enters the Arctic Basin through Fram Strait. Figure 1b, d, f indicates that sea ice concentration, sea surface heat flux, and MLD north of the Svalbard have undergone changes similar to the northern Barents Sea. In situ observations revealed a warming trend in the AW at Fram Strait^2^, and an increase in upward oceanic heat flux in the eastern Eurasian Basin^54^, both of which are associated with the ongoing Arctic sea ice decline^46,49^. Future changes in AW inflows in both the Fram Strait and Barents Sea branches could interact and influence the vertical temperature and salinity structures and ocean surface heat flux in the Arctic Basin together. Therefore, sustained observations along the pathways of both the AW branches are required to monitor and understand Arctic Ocean changes.

In summary, based on a reanalysis dataset and CMIP6 simulations, we find that the Arctic Ocean cooling machine will very likely expand poleward due to the atmospheric warming and this process will facilitate the poleward advance of the Arctic Atlantification, with poleward enhancement in ocean warming, winter sea ice decline, and upper ocean instability (Fig. 6). Despite the stabilization by the freshening trend in the upper ocean, the winter MLD in the northern Barents and Kara Seas will likely keep increasing for some decades in a future warming climate due to enhanced surface heat loss associated with the poleward expansion of the cooling machine. Under the SSP585 scenario, CMIP6 models predict that the northern Barents Sea will be ice-free in winter in ~2060. Ocean surface heat loss and winter MLD in the northern Barents Sea will increase until this period. The surface heat loss in the northern Kara Sea will reach its maximum before the end of the 21st century, so efficient surface cooling will possibly expand to the Arctic Basin afterward. Accordingly, the Arctic Ocean will be in a completely new regime with its deep basin receiving a large amount of ocean heat from the North Atlantic through two branches instead of only one as in the past. The poleward advance of the Arctic Ocean cooling machine and Arctic Atlantification in a warming world thus implies profound changes in the Arctic Ocean and climate that are very relevant to marine life and human activity.

## Methods

### Terminology

Arctic Basin refers to the central Arctic with bottom topography deeper than 500 m. Arctic Ocean refers to the Arctic Basin and its surrounding shelf seas, including Barents, Kara, Laptev, East Siberian, Chukchi, and Beaufort Seas.

### Reanalysis dataset

We used reanalysis dataset from the Ocean ReAnalysis System 5 (ORAS5)^55,56^ to investigate the changes in the air–sea fluxes and ocean conditions over the past four decades. The ORAS5 reanalysis dataset is from the ECMWF operational ensemble reanalysis–analysis system. It can be downloaded from https://icdc.cen.uni-hamburg.de/en/daten/reanalysis-ocean/easy-init-ocean/ecmwf-oras5.html. The observations of sea surface temperature, sea level anomaly, in situ temperature/salinity profiles, and sea ice concentration are assimilated in this system using NEMOVAR^57^. It includes five ensemble members and covers the period from 1979 onward. In this study, we used the mean results of the five ensemble members.

To evaluate the ORAS5, we compared its ocean temperature, salinity, and upper 100 m ocean heat content with long-term observations in the Barents Sea. Supplementary Figures 1, 17, and 18 show that their variabilities and long-term trends over the past 40 years in the Barents Sea are well reproduced by ORAS5. The evaluation shows that ORAS5 can be used for investigating climate change in the Barents Sea region. Due to the sparseness of in situ observations, we cannot evaluate the Kara Sea simulation in ORAS5 as done for the Barents Sea. Instead, we compared four widely used reanalysis products for ocean surface heat flux, the key diagnostic studied in this paper (Supplementary Fig. 19). We noticed that the past change in ocean surface heat flux in ORAS5 has a spatial pattern very similar with those in SODA3.4.2 (ref. ^58^) and ERA5 (ref. ^59^). The surface heat flux from SODA3.4.2 and ERA5 can be downloaded from https://www2.atmos.umd.edu/~ocean/index_files/soda3_readme.htm and https://cds.climate.copernicus.eu/#!/home, respectively. In our study, we also used the surface air temperature and surface heat flux from ERA-Interim^60^, which can be downloaded from https://apps.ecmwf.int/datasets/data/interim-full-daily/levtype=sfc/.

### CMIP6 dataset

The first realizations of 12 CMIP6 coupled models are used (Supplementary Table 1), including CanESM5, CESM2, CESM2-WACCM, CNRM-CM6-1, EC-Earth3-Veg, FIO-ESM-2-0, GFDL-CM4, IPSL-CM6A-LR, MPI-ESM1-2-HR, MPI-ESM1-2-LR, NorESM2-LM, and UKESM1-0-LL. The simulations for the period from 1979 to 2014 are from their historical experiments. The future projections in the CMIP6 are driven by external forcings based on the SSP framework^61^, which was established by the climate change research community, in order to facilitate the integrated analysis of future climate impacts, vulnerabilities, adaptation, and mitigation^62^. In this study, we use the SSP585 experiments. The SSP585 scenario is based on the narrative of fossil-fueled development with high challenges to mitigation and low challenges to adaptation^62^. It represents the high end of the range of future pathways with an effective radiative forcing of 8.5 Wm^−2^ in 2100 (ref. ^61^). CMIP6 data can be downloaded from https://esgf-index1.ceda.ac.uk/projects/cmip6-ceda/.

To evaluate CMIP6 performance, we compared CMIP6-simulated cold season sea surface heat flux, sea ice concentration, and MLD with reanalysis dataset during 1979–2018. Supplementary Figure 20 shows that the simulated climatological sea surface heat flux, sea ice concentration, and MLD from CMIP6 MMM results compare well with the reanalysis and observations. The observed increasing trends in ocean temperature and heat content are also well reproduced in the CMIP6 MMM (Supplementary Figs. 3 and 4). In the CMIP6 MMM, the BSW in the NEBS has warming and salinification trends over the past four decades, but the associated changes in density are small due to the compensating effects of salinity and temperature on density (Supplementary Fig. 21). In the southwestern Barents Sea, the ocean bottom density in the CMIP6 MMM decreases over the past four decades due to the dominating effect of the increase in temperature. These results are consistent with BSW observations^16^.

We found that the long-term trends in sea ice concentration, MLD, and sea surface heat flux are largely reproduced in the CMIP6 MMM, although there is underestimation in these trends compared to the reanalysis dataset and observations (Fig. 2). If the underestimation remains in the future projection simulations, the implication is that the progress of the poleward expansion of the cooling machine and the strengthening of Arctic Atlantification are underestimated in the simulations.

### Observation data

Several observation datasets are also used in this study. The satellite-observed sea ice concentration dataset is from National Snow and Ice Data Center (http://nsidc.org/data/seaice/), which is retrieved using the National Aeronautics and Space Administration (NASA) team algorithm^63^. Ocean temperature and salinity observations used for reanalysis dataset validation in Supplementary Fig. 1 are from https://ocean.ices.dk/iroc/. Temperature and salinity atlas of the Barents Sea^64^ and observed northern Barents Sea ocean heat content^41^ were also used to evaluate the reanalysis dataset and CMIP6 MMM in Supplementary Figs. 4, 17, and 18. The 0 °C surface isotherm from WOA13 climatology^65^ was used to indicate the boundary between southwestern and northern Barents Sea in Fig. 1, and Supplementary Figs. 2 and 20. It also represents the boundary between ice-free and ice-covered regions in the climatology. The WOA13 climatology can be downloaded from https://www.nodc.noaa.gov/OC5/woa13/. The long-term series of sea surface temperature of HadISST1 (ref. ^66^) from Met Office Hadley Centre was used to show its linear trend, which is available from https://www.metoffice.gov.uk/hadobs/hadisst/.

## Supplementary information

Supplementary Information

## Data Availability

All the data used in this research are freely available to the public and can be downloaded through the links detailed in the “Methods” section.

## Code availability

All code used to produce the figures are available from the corresponding author on request.
