# Supplementary material for: The poleward enhanced Arctic Ocean cooling machine in a warming climate
Source: Nat Commun. 2021 May 20;12:2966. doi: 10.1038/s41467-021-23321-7 (PMC8137913; doi:10.1038/s41467-021-23321-7)
Supplement: Supplementary file 1 — Supplementary Information [file 41467_2021_23321_MOESM1_ESM.pdf]

The poleward enhanced Arctic Ocean cooling machine in a warming climate

Qi Shu[1, 2, 3], Qiang Wang[4*], Zhenya Song[1, 2, 3], Fangli Qiao[1, 2, 3*]

[1]First Institute of Oceanography, and Key Laboratory of Marine Science and Numerical Modeling, Ministry of Natural Resources, Qingdao, China

[2]Laboratory for Regional Oceanography and Numerical Modeling, Pilot National Laboratory for Marine Science and Technology, Qingdao, China

[3]Shandong Key Laboratory of Marine Science and Numerical Modeling, Qingdao, China

[4]Alfred Wegener Institute Helmholtz Centre for Polar and Marine Research (AWI), Bremerhaven, Germany

*Corresponding authors: Fangli Qiao (qiaofl@fio.org.cn); Qiang Wang (Qiang.Wang@awi.de)

Supplementary

Supplementary Table 1. CMIP6 models used in this study.

No.	Model name	Institution ID	Data DOI
1	CanESM5	CCCma	http://doi.org/10.22033/ESGF/CMIP6.3610<br>http://doi.org/10.22033/ESGF/CMIP6.3696
2	CESM2	NCAR	http://doi.org/10.22033/ESGF/CMIP6.7627<br>http://doi.org/10.22033/ESGF/CMIP6.7768
3	CESM2-WACCM	NCAR	http://doi.org/10.22033/ESGF/CMIP6.10071<br>http://doi.org/10.22033/ESGF/CMIP6.10115
4	CNRM-CM6-1	CNRM-CERFACS	http://doi.org/10.22033/ESGF/CMIP6.4066<br>http://doi.org/10.22033/ESGF/CMIP6.4224
5	EC-Earth3-Veg	EC-Earth-Consortium	http://doi.org/10.22033/ESGF/CMIP6.4706<br>http://doi.org/10.22033/ESGF/CMIP6.4914
6	FIO-ESM-2-0	FIO-QLNM	http://doi.org/10.22033/ESGF/CMIP6.9199<br>http://doi.org/10.22033/ESGF/CMIP6.9214
7	GFDL-CM4	NOAA-GFDL	http://doi.org/10.22033/ESGF/CMIP6.8594<br>http://doi.org/10.22033/ESGF/CMIP6.9268
8	IPSL-CM6A-LR	IPSL	http://doi.org/10.22033/ESGF/CMIP6.5195<br>http://doi.org/10.22033/ESGF/CMIP6.5271
9	MPI-ESM1-2-HR	MPI-M	http://doi.org/10.22033/ESGF/CMIP6.6594<br>http://doi.org/10.22033/ESGF/CMIP6.4403
10	MPI-ESM1-2-LR	MPI-M	http://doi.org/10.22033/ESGF/CMIP6.6595<br>http://doi.org/10.22033/ESGF/CMIP6.6705
11	NorESM2-LM	NCC	http://doi.org/10.22033/ESGF/CMIP6.8036<br>http://doi.org/10.22033/ESGF/CMIP6.8319
12	UKESM1-0-LL	MOHC	http://doi.org/10.22033/ESGF/CMIP6.6113<br>http://doi.org/10.22033/ESGF/CMIP6.6405

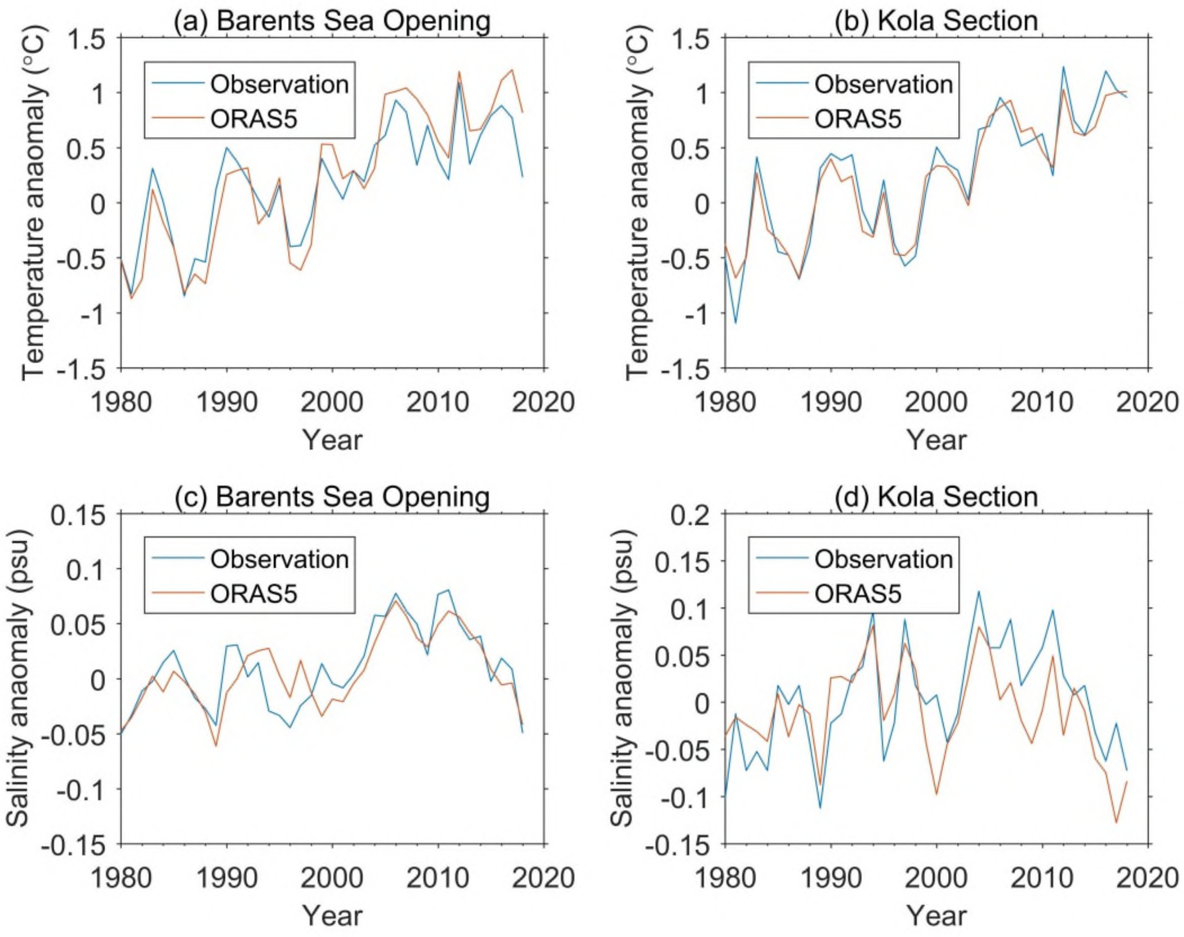

Supplementary Fig. 1. Evaluation of temperature and salinity in reanalysis against in-situ observations. The comparison of (a-b) temperature and (c-d) salinity anomalies between reanalysis (ORAS5) and observations. Anomalies are referenced to the mean over the period 1979-2008. The locations of Barents Sea Opening and Kola Section observations are along 20ºE and 33.5ºE, respectively.

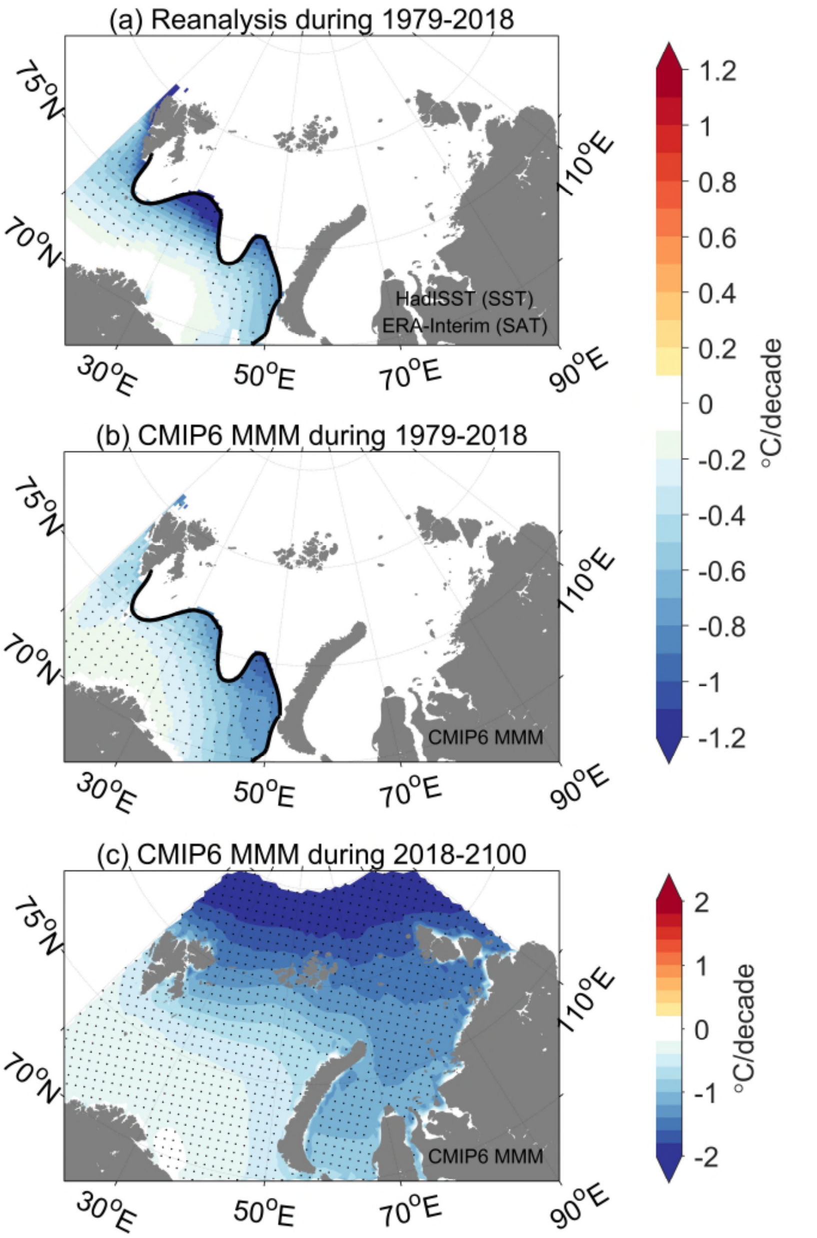

Supplementary Fig. 2. The different warming rates of sea surface temperature and surface air temperature. Linear trends of the difference between sea surface temperature (SST) and surface air temperature (SAT) in the cold season during 1979/80-2017/18 based on (a) HadISST observations, ERA-Interim reanalysis and (b) CMIP6 MMM. (c) is same as (b) but for 2018/19-2099/2100. The black lines in (a) and (b) are the 0 ºC surface isotherm based on climatology from WOA13, which can represent the boundary between the southwestern and northern Barents Sea. In (c) the results in the entire Barents and Kara

Seas are shown, but the trend is relevant to ocean surface heat loss only in ice-free areas which evolve with time, because sea ice inhibits the air-sea heat exchange.

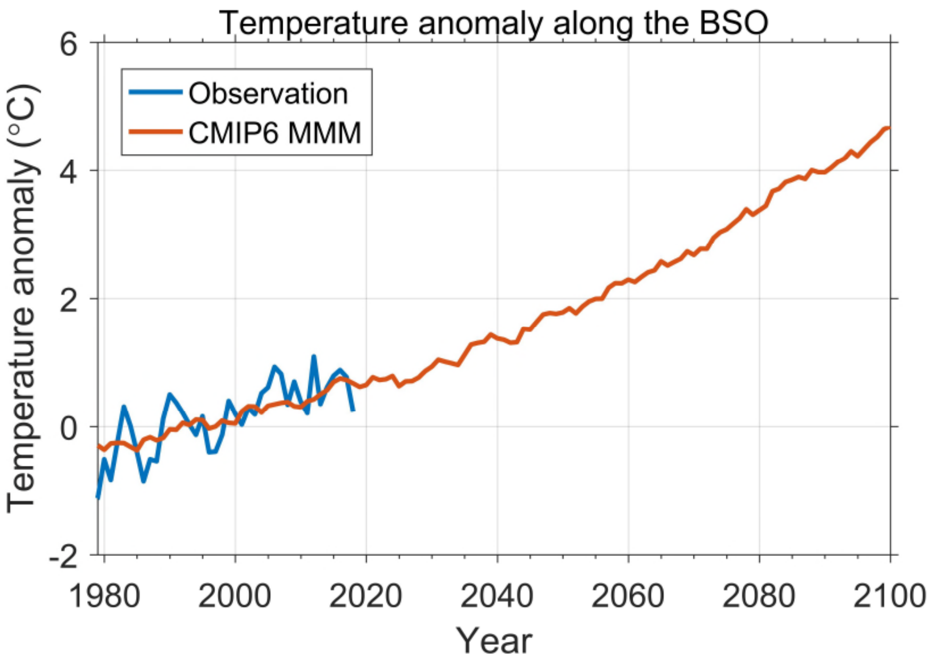

Supplementary Fig. 3. Ocean temperature anomalies along the Barents Sea Opening (BSO). The blue line shows the observation, and the red line is for the CMIP6 historical and SSP585 MMM.

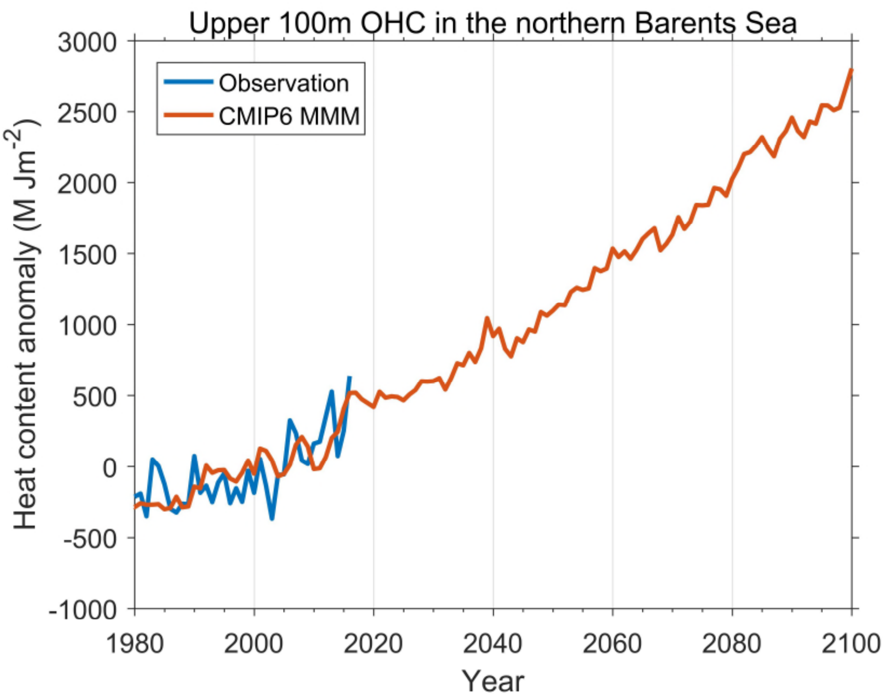

Supplementary Fig. 4. Evaluation of the trend in CMIP6 MMM ocean heat content (OHC) in the Northern Barents Sea. The upper 100 m ocean heat content anomaly in late summer and early autumn (August-September) in the northern Barents Sea from the observation[41] (blue) and CMIP6 MMM (red).

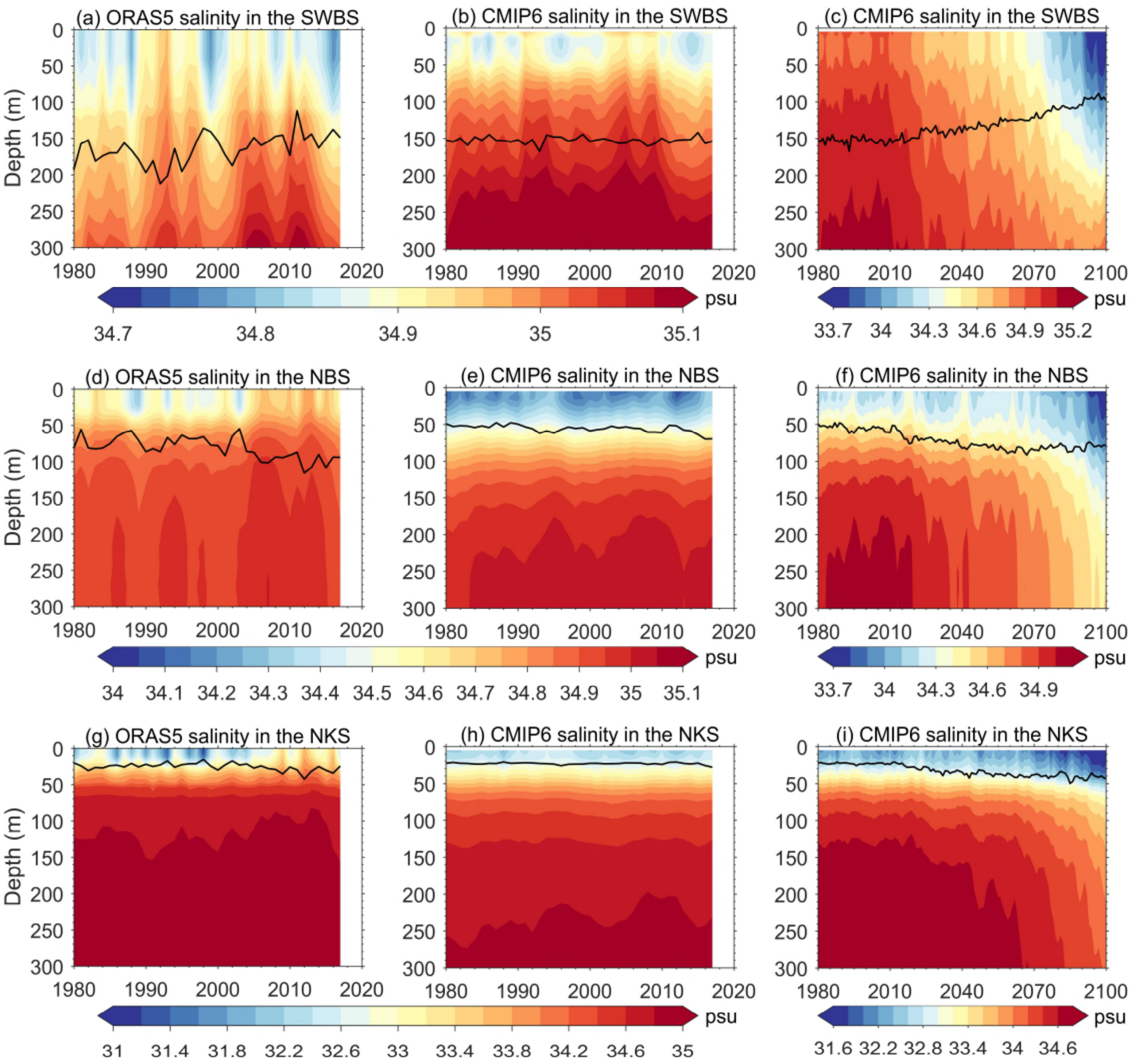

Supplementary Fig. 5. The changes of salinity in the southwestern Barents Sea, northern Barents and Kara Seas. (a-c) Depth-time plots of cold season salinity averaged in the southwestern Barents Sea (SWBS, the red box shown in Fig. 2a) in the reanalysis (ORAS5) and CMIP6 MMM. (d-f) The same as panels (a-c) but for the northern Barents Sea (NBS, the black box shown in Fig. 2a). (g-i) The same as panels (a-c) but for the northern Kara Sea (NKS, the magenta box shown in Fig. 2a). The black lines show the MLD averaged in the respective boxes.

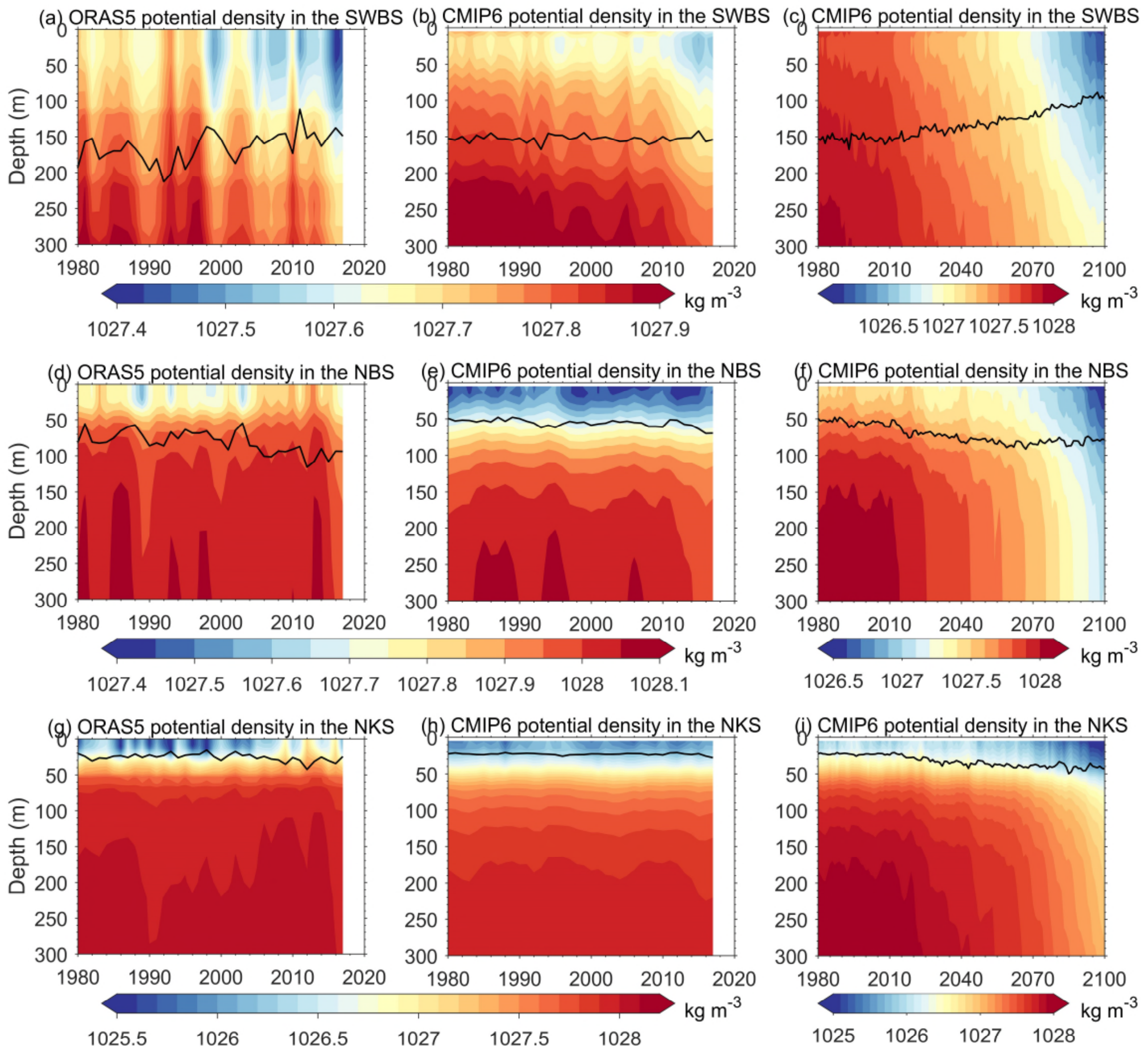

Supplementary Fig. 6. The changes of potential density in the southwestern Barents Sea, northern Barents and Kara Seas. (a-c) Depth-time plots of cold season potential density averaged in the southwestern Barents Sea (SWBS, the red box shown in Fig. 2a) in the reanalysis (ORAS5) and CMIP6 MMM. (d-f) The same as panels (a-c) but for the northern Barents Sea (NBS, the black box shown in Fig. 2a). (g-i) The same as panels (a-c) but for the northern Kara Sea (NKS, the magenta box shown in Fig. 2a). The black lines show the MLD averaged in the respective boxes.

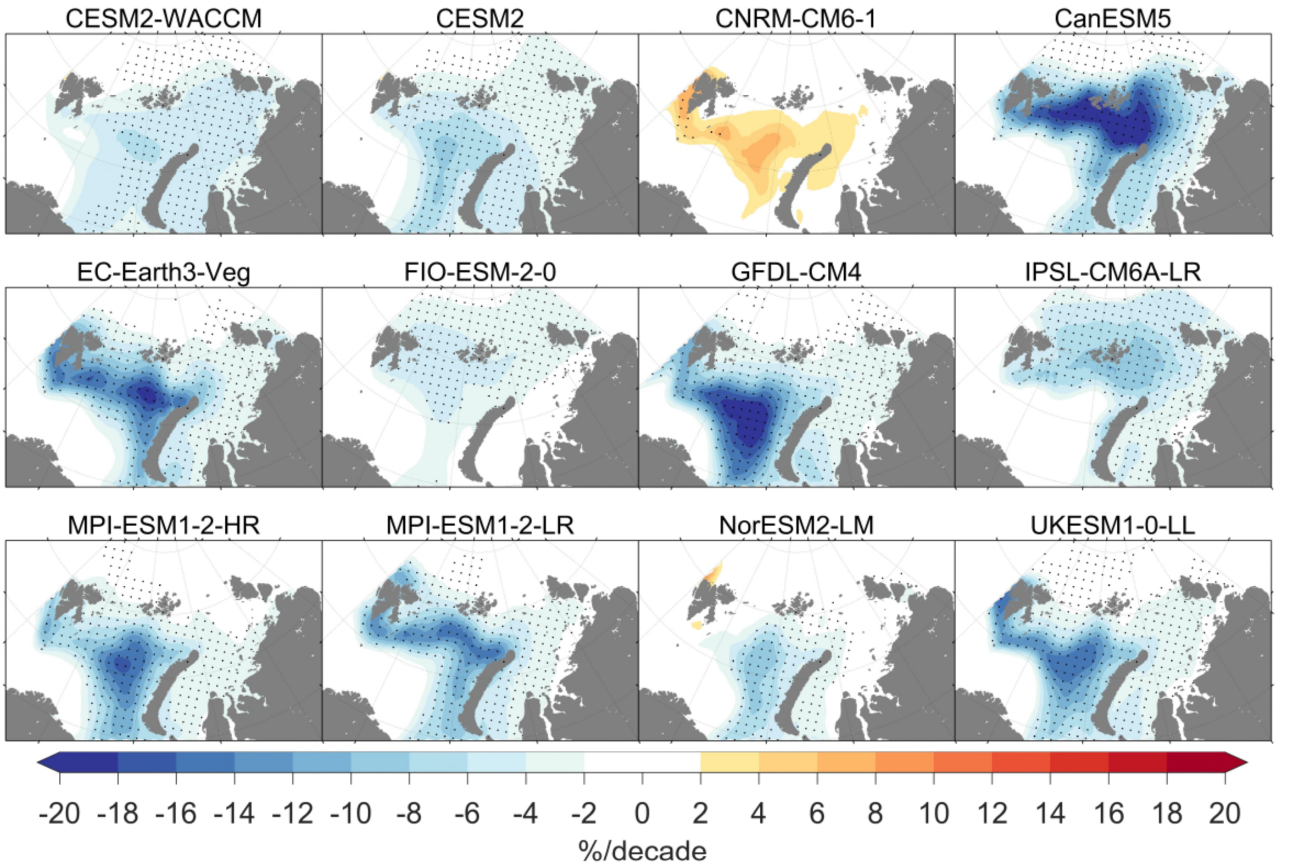

Supplementary Fig. 7. Past trends of sea ice concentration in CMIP6 models. Linear trend of sea ice concentration in the cold season (October-March) over the period 1979/80-2017/18 in each CMIP6 model. Dots indicate that linear trends exceed the 95% confidence level.

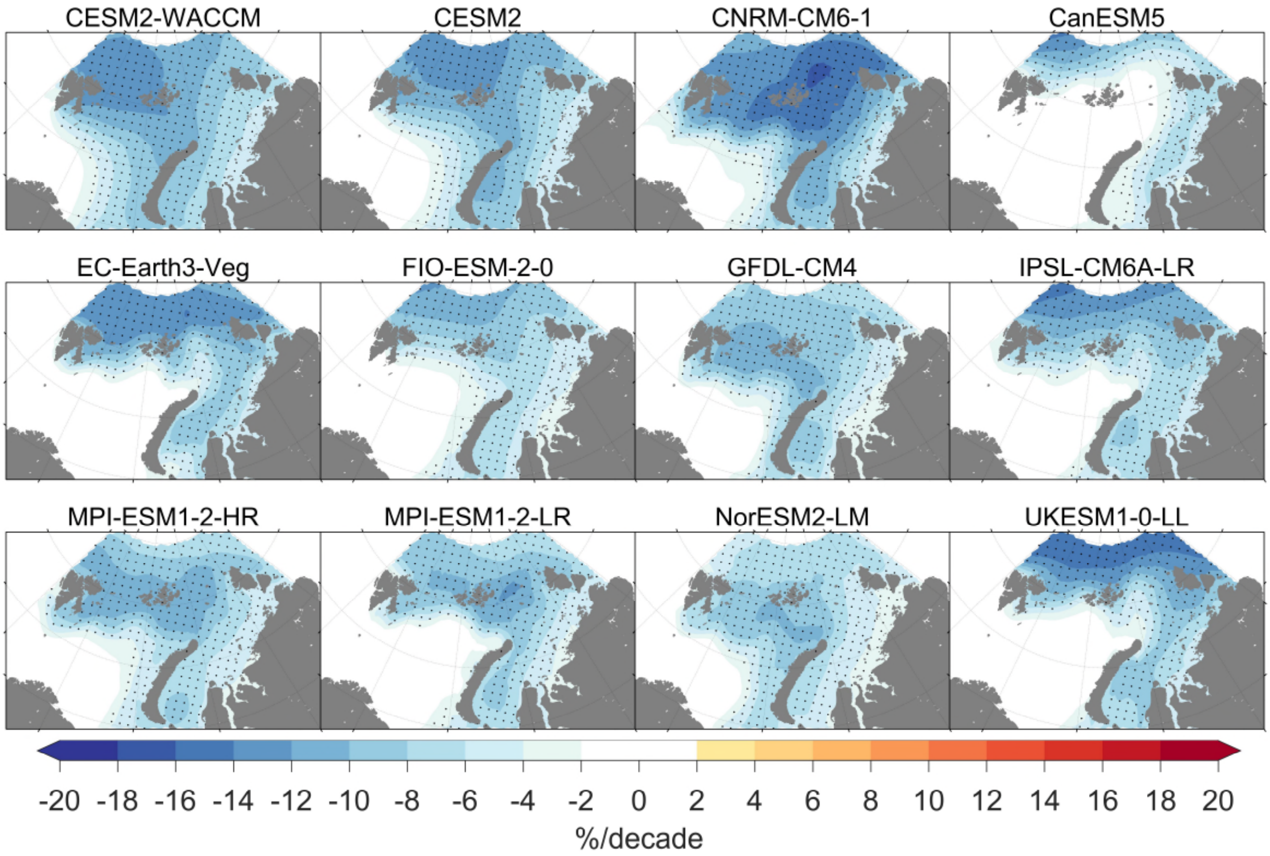

Supplementary Fig. 8. Future trends of sea ice concentration in CMIP6 models. Linear trend of sea ice concentration in the cold season (October-March) over the period 2018/19-2099/2100 in each CMIP6 model. Dots indicate that linear trends exceed the 95% confidence level.

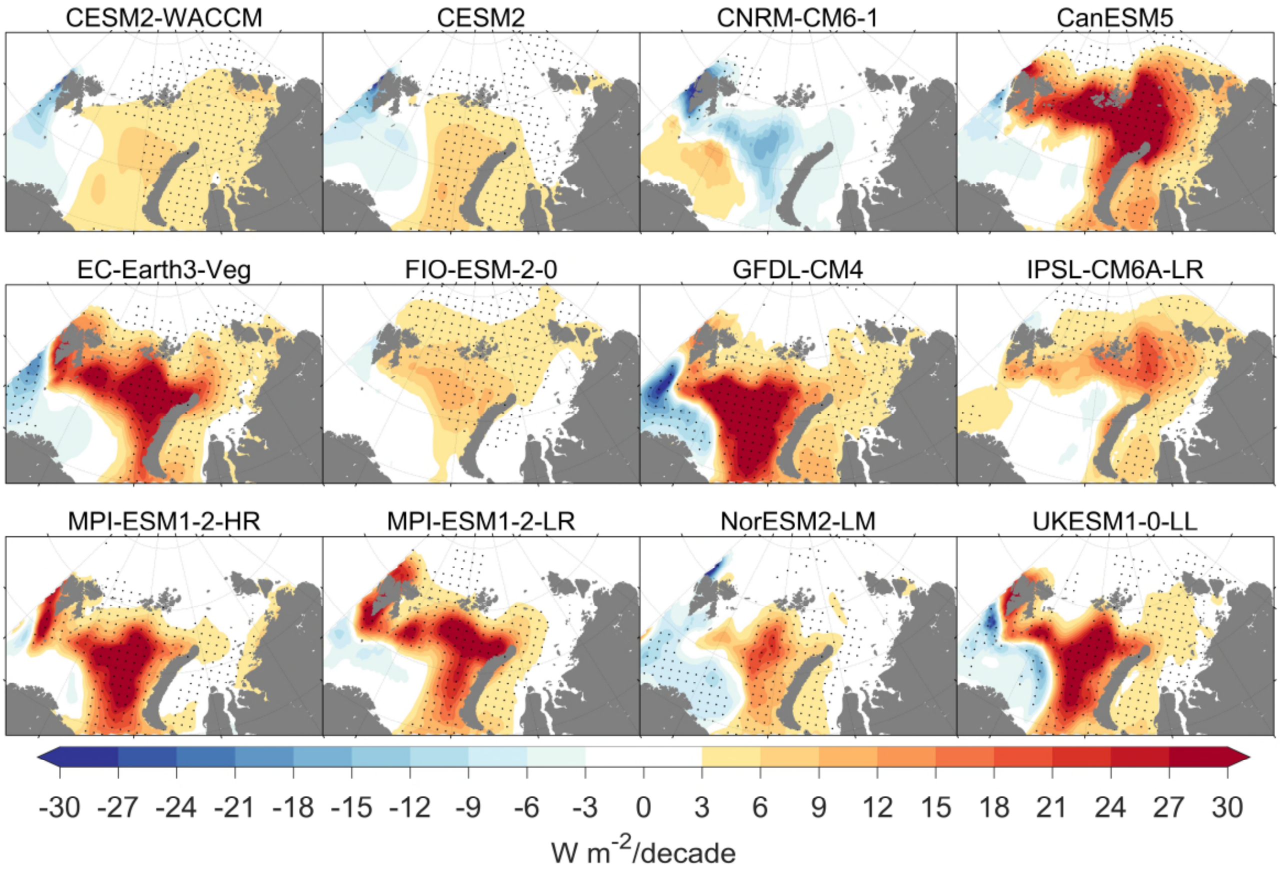

Supplementary Fig. 9. Past trends of sea surface heat flux in CMIP6 models. Linear trend of sea surface heat flux in the cold season (October-March) over the period 1979/80-2017/18 in each CMIP6 model. Dots indicate that linear trends exceed the 95% confidence level. Upward sea surface heat flux is positive.

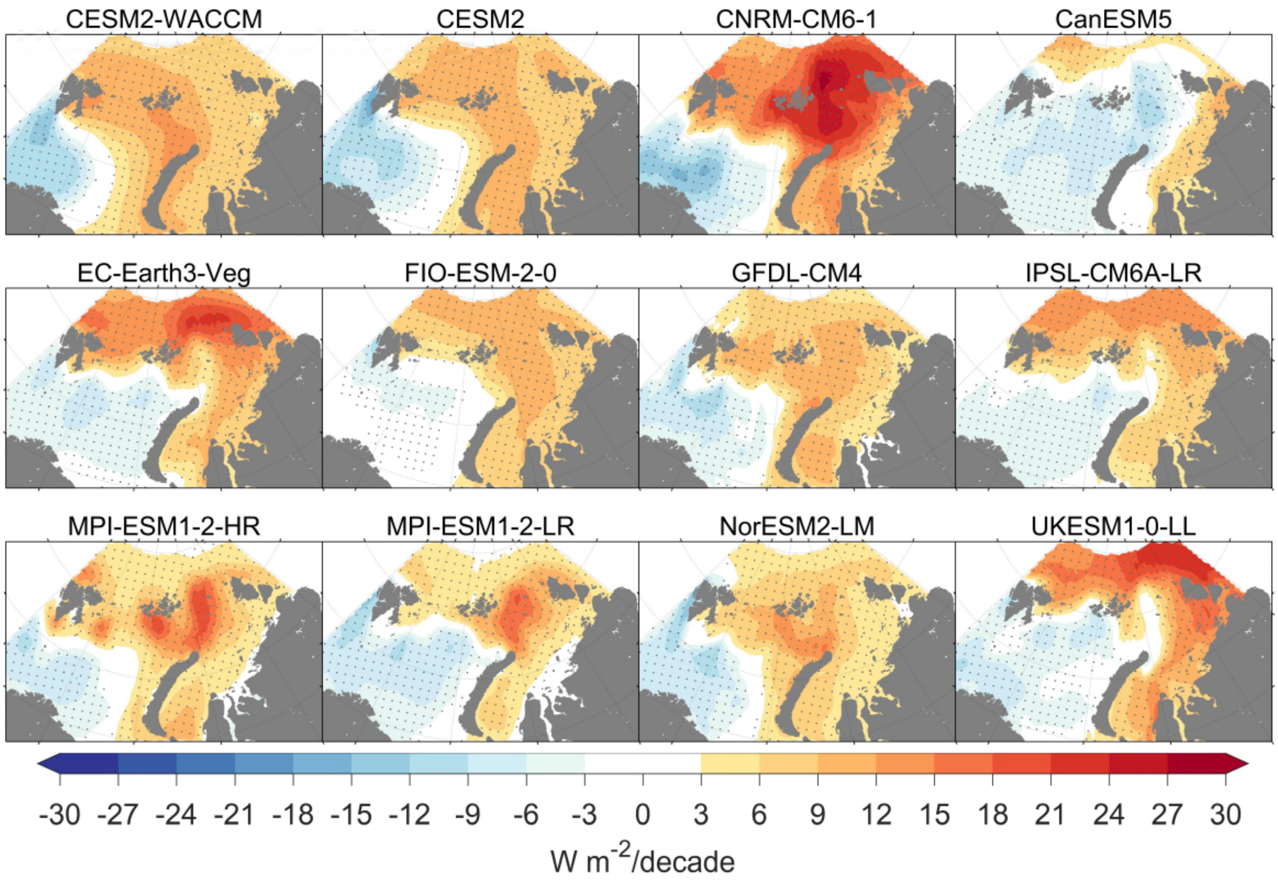

Supplementary Fig. 10. Future trends of sea surface heat flux in CMIP6 models. Linear trend of sea surface heat flux in the cold season (October-March) over the period 2018/19-2099/2100 in each CMIP6 model. Dots indicate that linear trends exceed the 95% confidence level. Upward sea surface heat flux is positive.

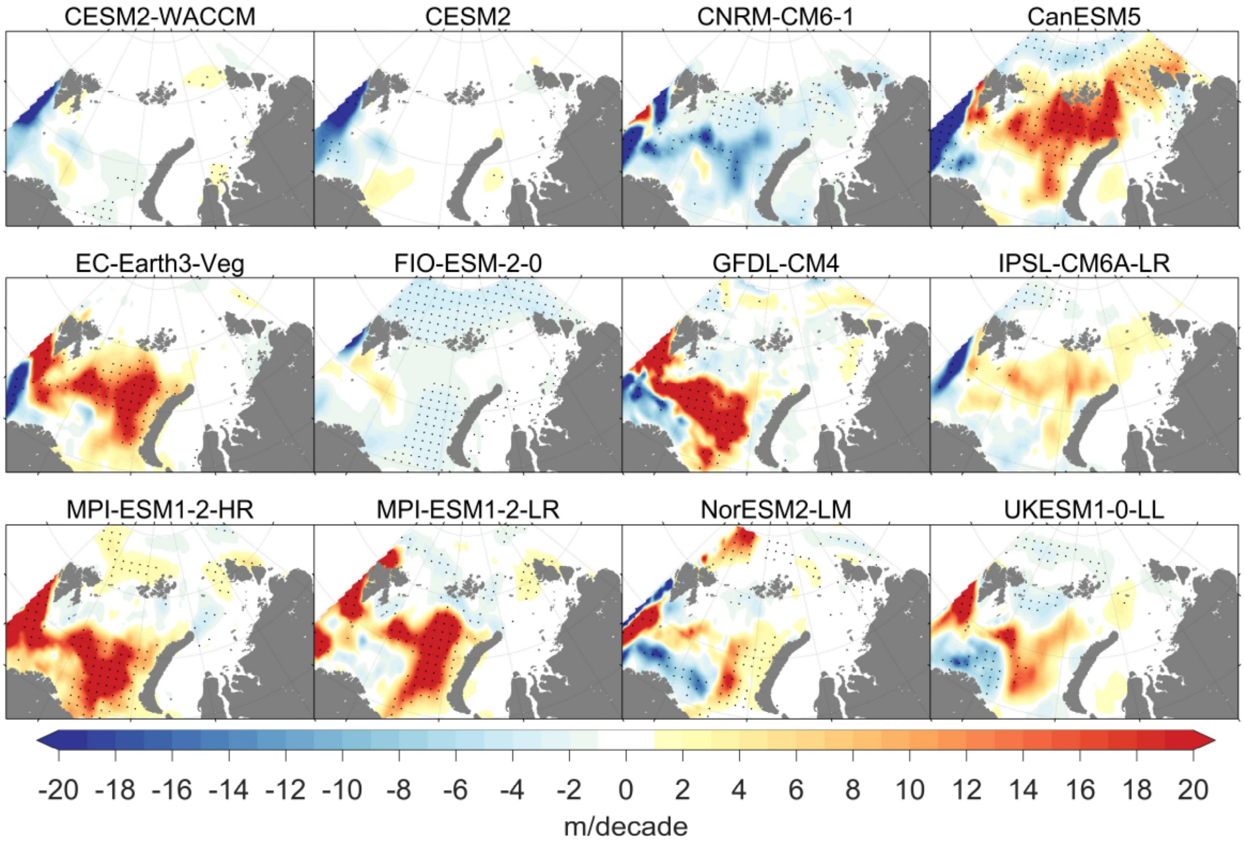

Supplementary Fig. 11. Past trends of mixed layer depth in CMIP6 models. Linear trend of mixed layer depth in the cold season (October-March) over the period 1979/80-2017/18 in each CMIP6 model. Dots indicate that linear trends exceed the 95% confidence level.

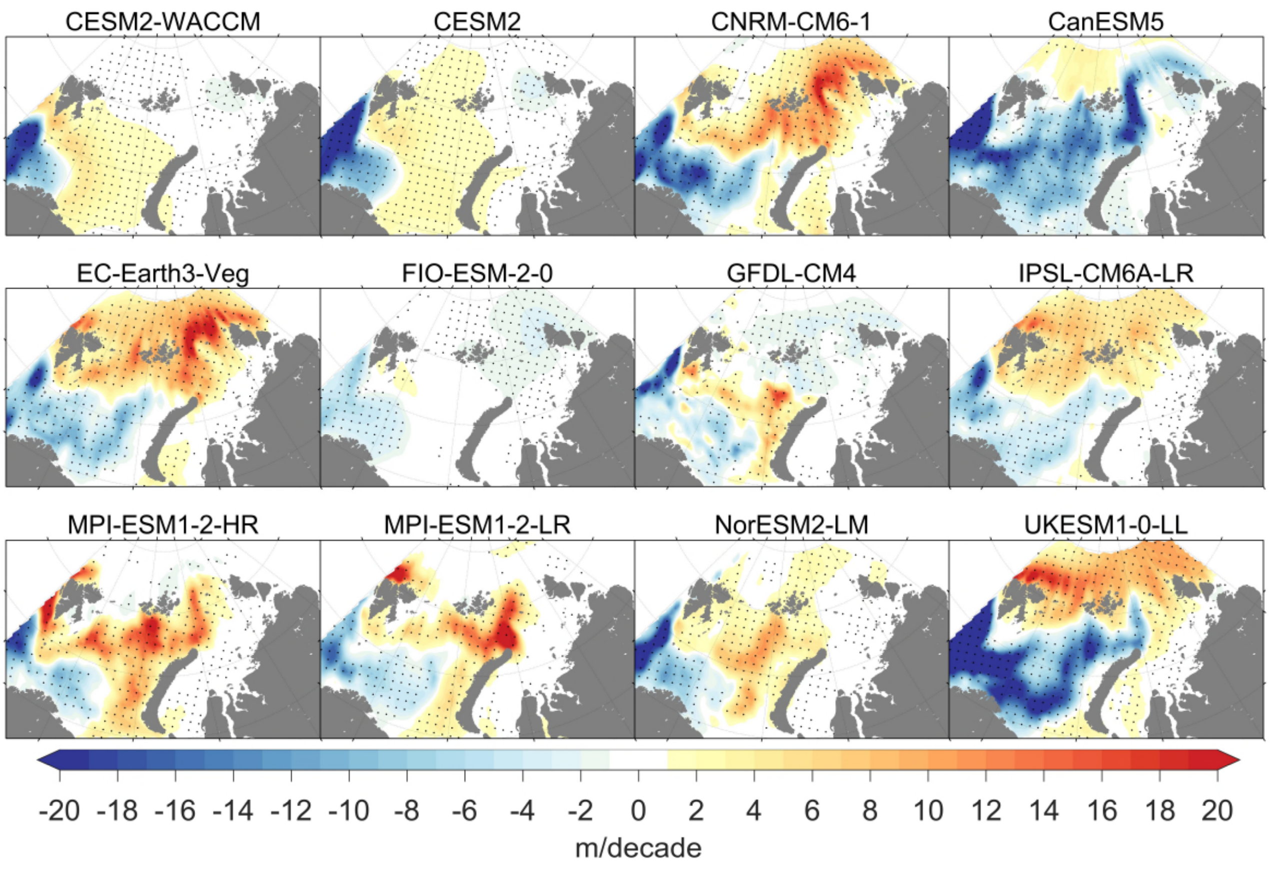

Supplementary Fig. 12. Future trends of mixed layer depth in CMIP6 models. Linear trend of mixed layer depth in the cold season (October-March) over the period 2018/19-2099/2100 in each CMIP6 model. Dots indicate that linear trends exceed the 95% confidence level.

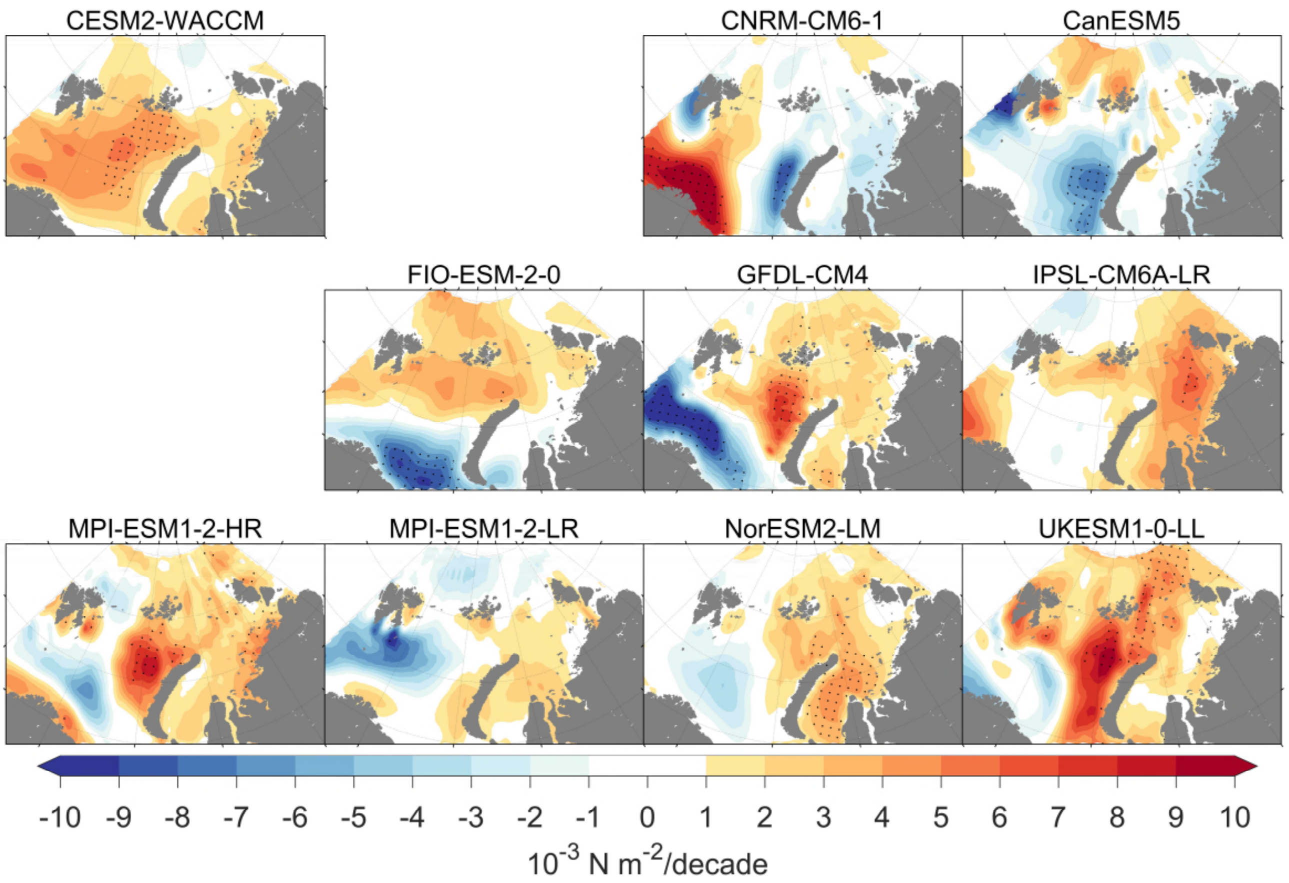

Supplementary Fig. 13. Past trends of surface stress in CMIP6 models. Linear trend of surface stress in the cold season (October-March) over the period 1979/80-2017/18 in each CMIP6 model. Dots indicate that linear trends exceed the 95% confidence level.

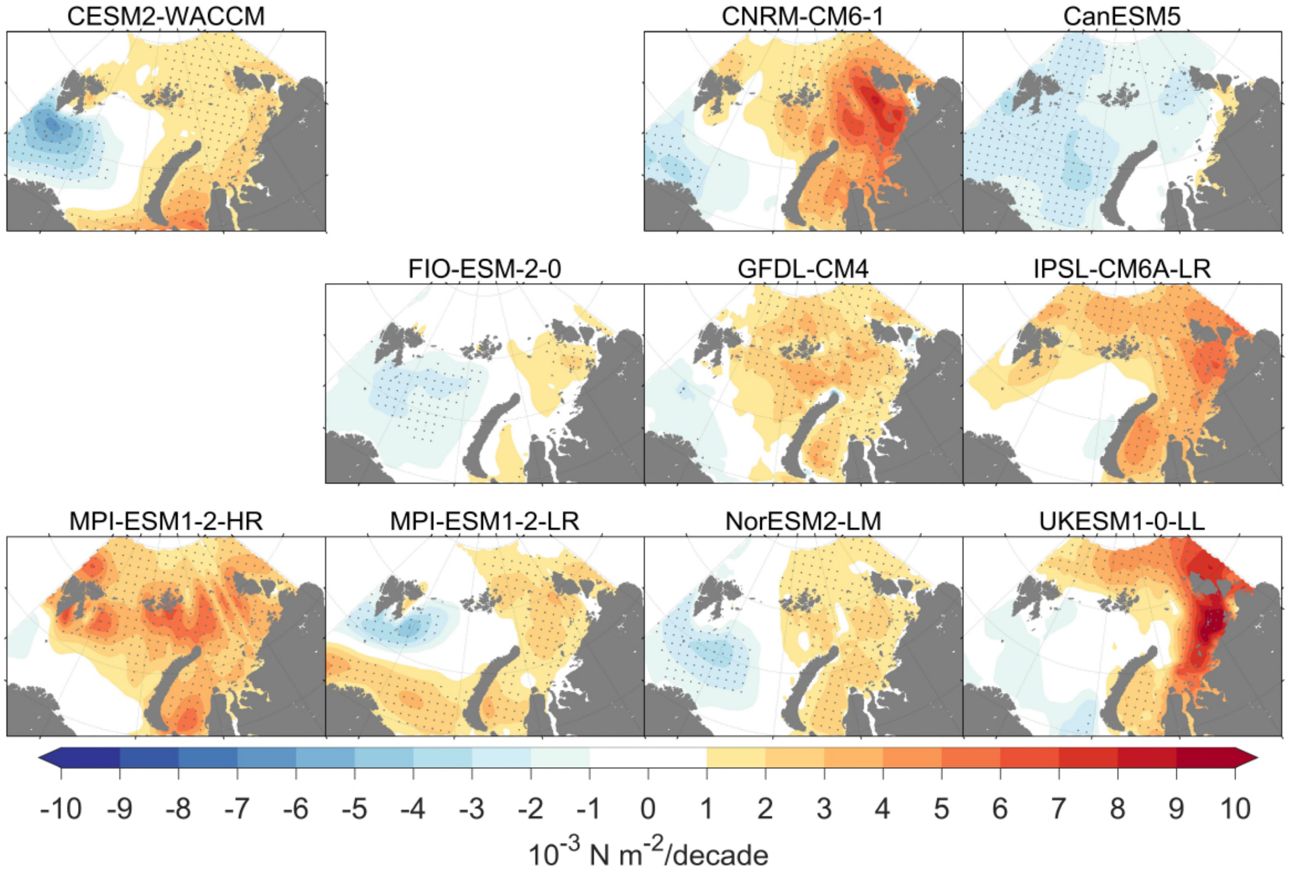

Supplementary Fig. 14. Future trends of surface stress in CMIP6 models. Linear trend of surface stress in the cold season (October-March) over the period 2018/19-2099/2100 in each CMIP6 model. Dots indicate that linear trends exceed the 95% confidence level.

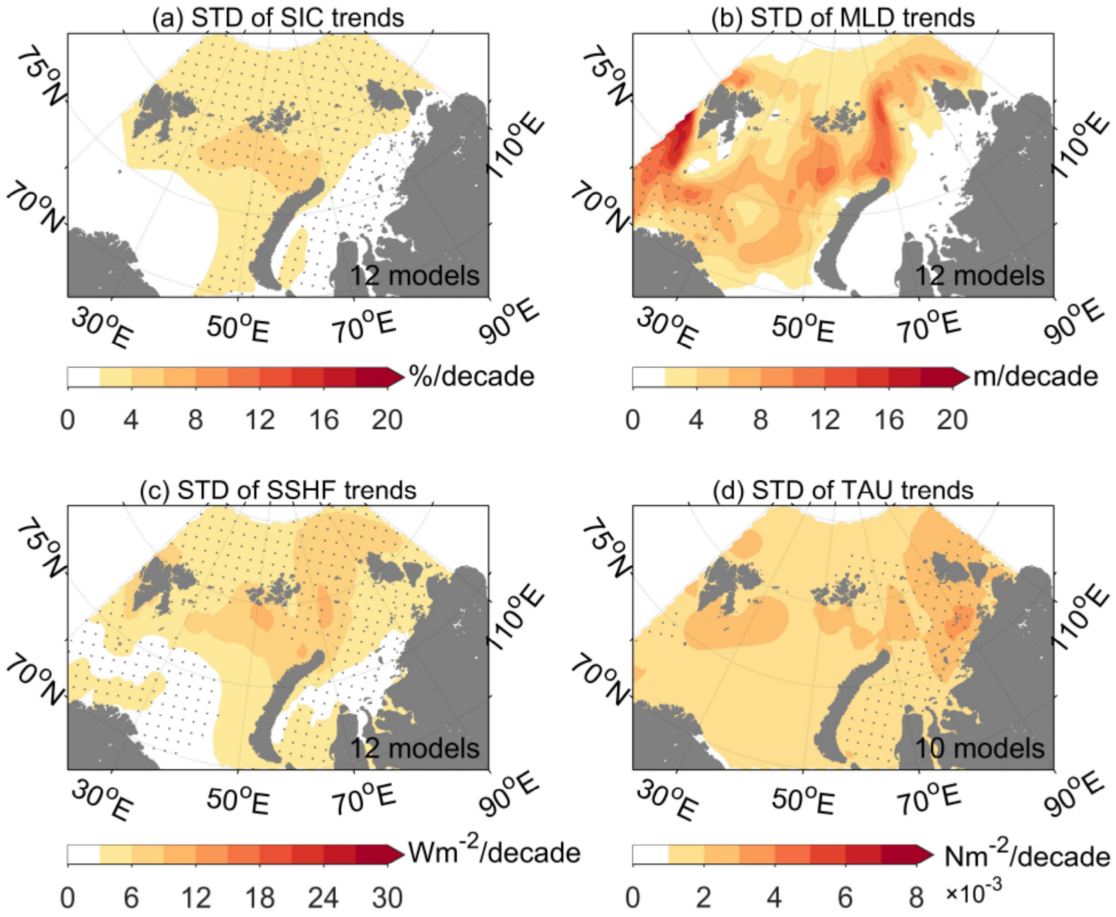

Supplementary Fig. 15. CMIP6 inter-model spread (one standard deviation, STD) of linear trends in the cold season during 2018/19 to 2099/2100. (a) Sea ice concentration (SIC), (b) mixed layer depth (MLD), (c) sea surface heat flux (SSHF), and (d) surface stress (TAU). Dots indicate that the inter-model spreads are smaller than the absolute values of the linear trends.

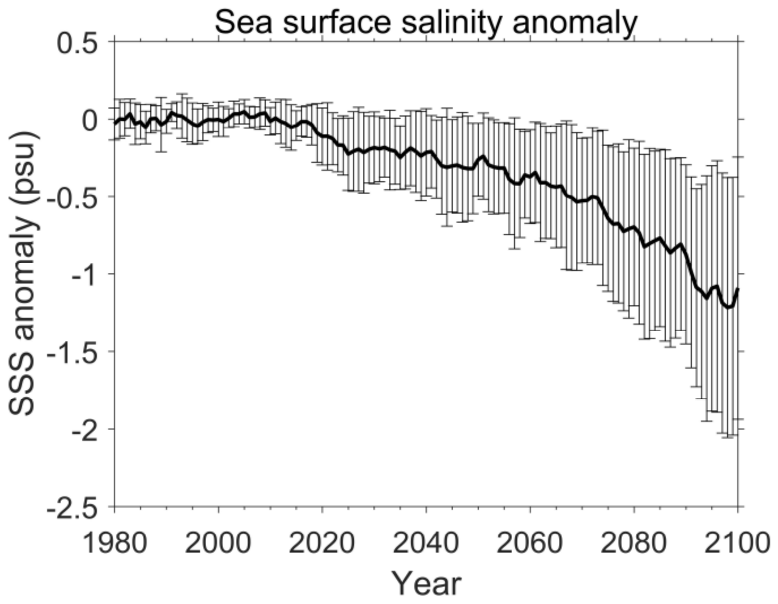

Supplementary Fig. 16. Sea surface salinity (SSS) anomaly in the southwestern Barents Sea based on CMIP6 models. The black line is the multi-model mean result. Error bars denote one standard deviation, which represents the inter-model spread. Anomalies are referenced to the mean of the 1980s.

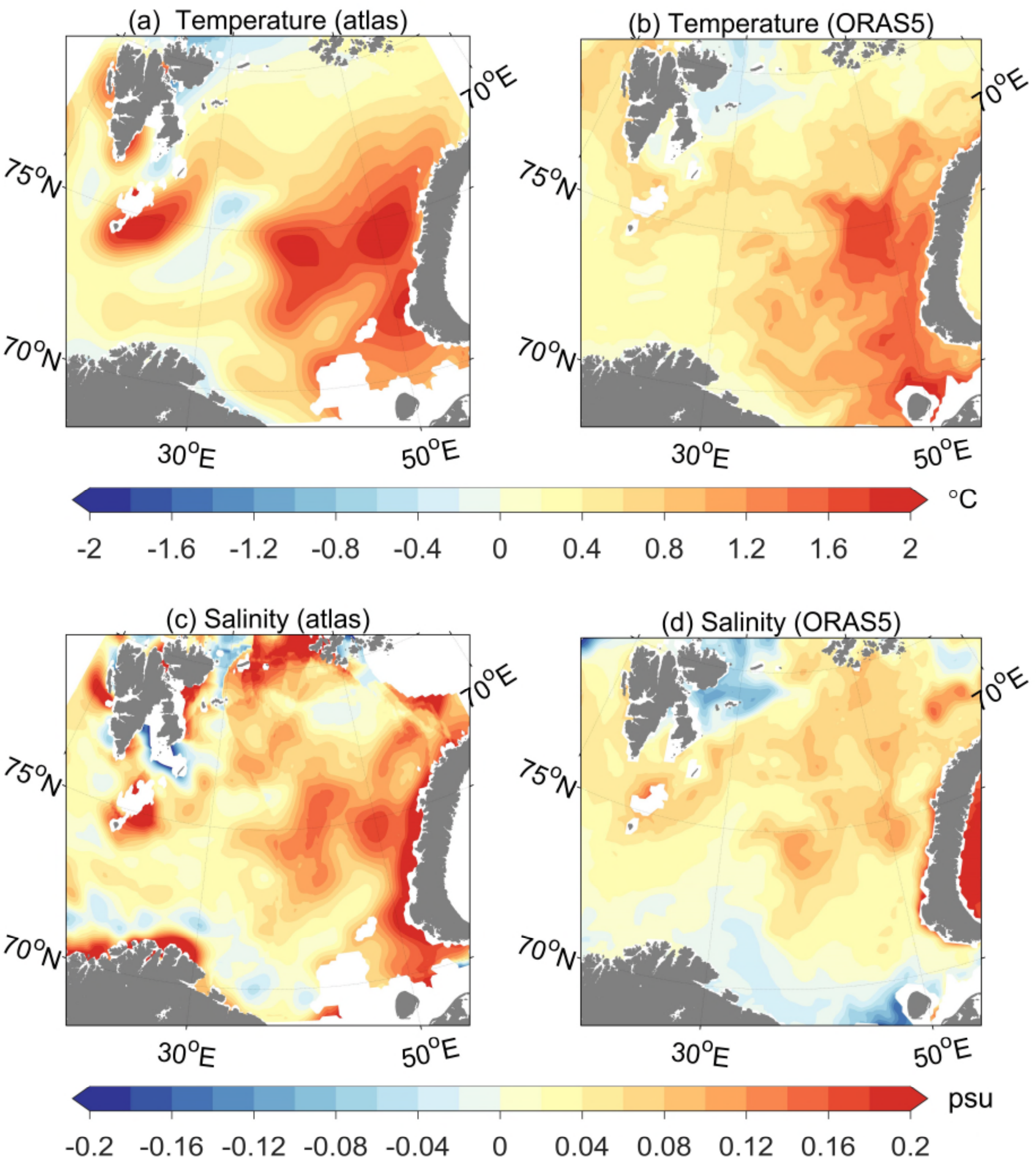

Supplementary Fig. 17. Evaluation of temperature and salinity in reanalysis against Barents Sea temperature and salinity atlas. (a and b) Temperature and (c and d) salinity differences at the depth of 50 m in autumn (August-October) between 2007-2016 and 1997–2006 in the (a and c) atlas[64] and (b and d) reanalysis (ORAS5).

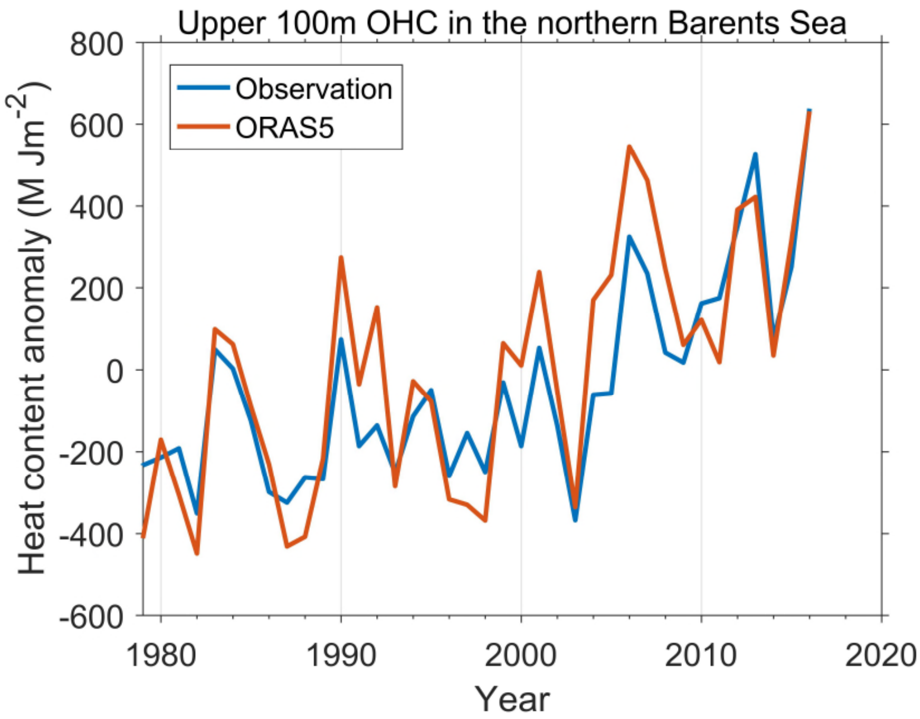

Supplementary Fig. 18. Evaluation of ocean heat content (OHC) in the Northern Barents Sea in the reanalysis. The upper 100 m ocean heat content (OHC) anomaly in late summer and early autumn (August-September) in the northern Barents Sea from the observation[41] (blue) and ORAS5 reanalysis (red).

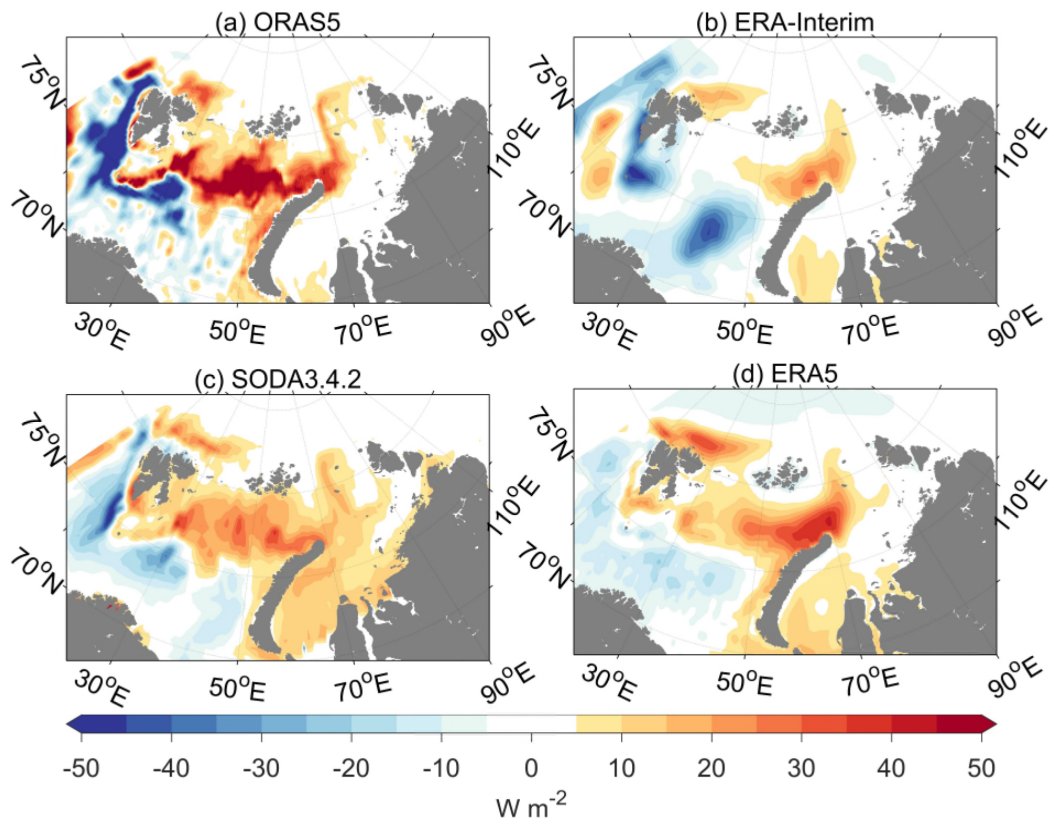

Supplementary Fig. 19. Intercomparison of the changes of ocean surface heat flux in four widely used reanalysis products. Differences in ocean surface heat flux between the warm period 2004-2018 and the cold period 1985-1999 based on (a) ORAS5, (b) ERA-Interim[60], (c) SADA3.4.2[58], and (d) ERA5[59].

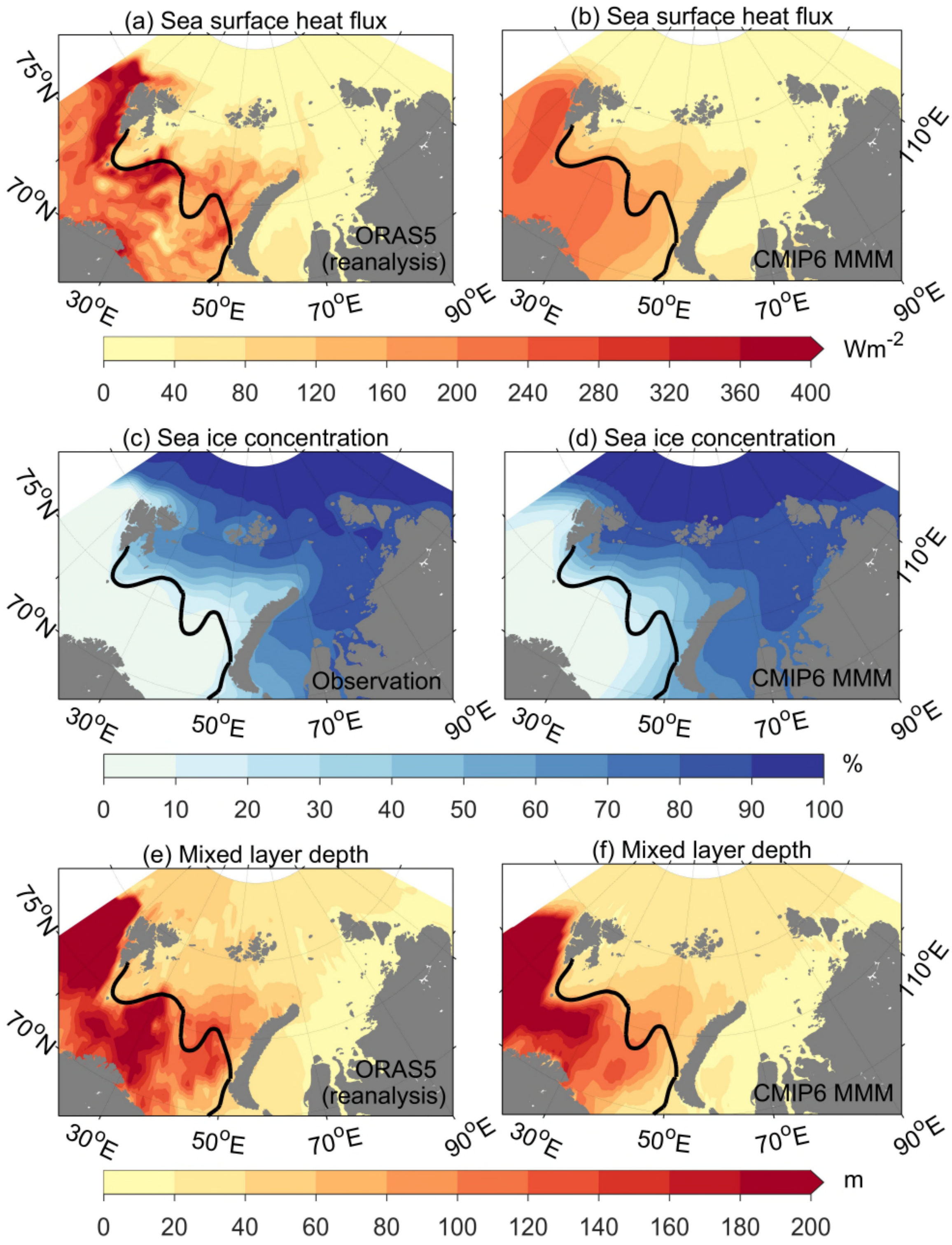

Supplementary Fig. 20. Evaluation of CMIP6 performance in simulating climatology. The comparison of (a-b) sea surface heat flux, (c-d) sea ice concentration, and (e-f) mixed layer depth in the cold season between CMIP6 MMM and observations and the reanalysis

dataset (ORAS5) during 1979/80-2017/18. The black lines are the 0 $^{\circ}$C surface isotherm based on climatology from WOA13, which can represent the boundary between the southwestern and northern Barents Sea. Upward sea surface heat flux is positive.

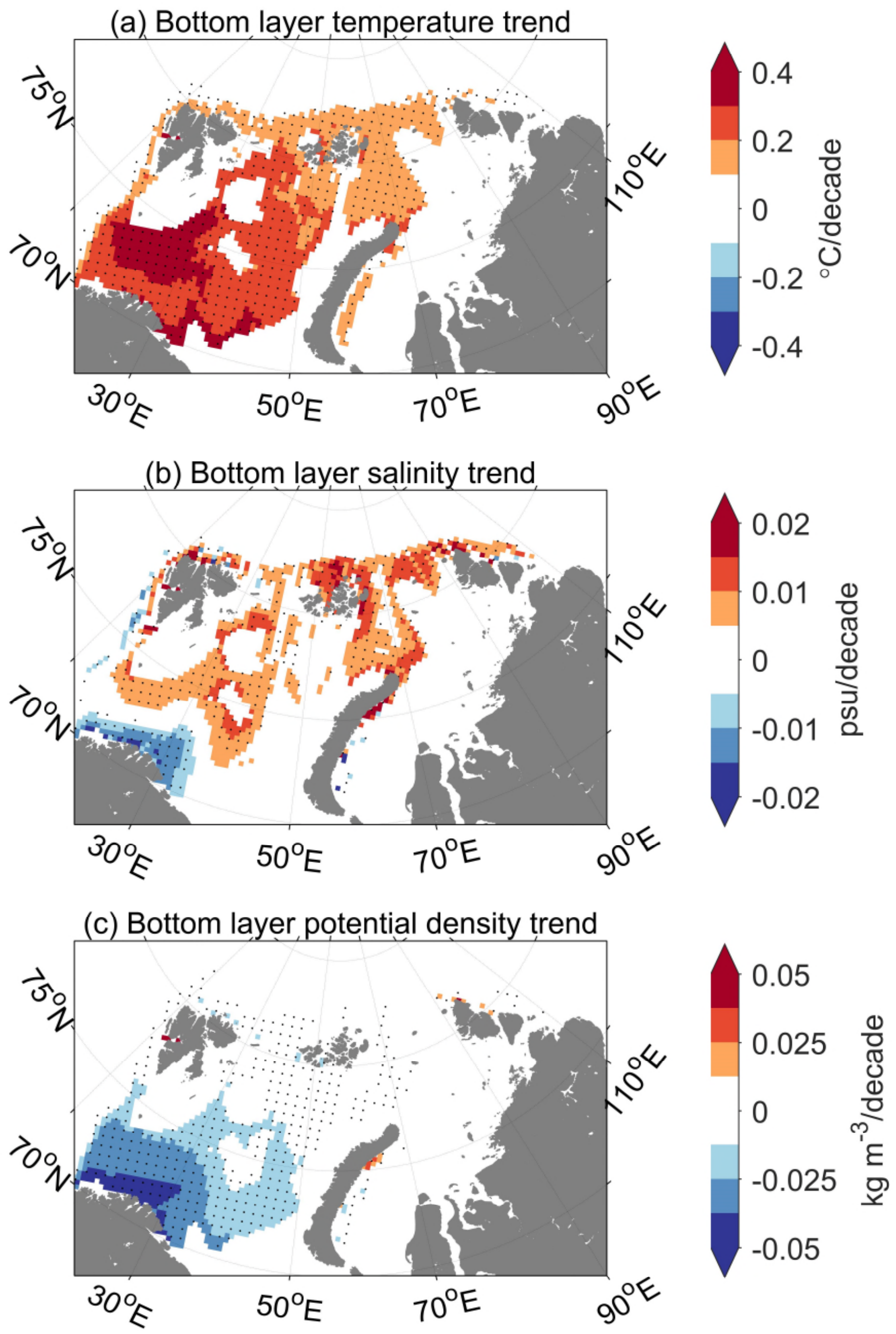

Supplementary Fig. 21. Past changes in bottom water. Linear trends of CMIP6 MMM bottom layer (a) temperature, (b) salinity, and (c) potential density referenced to surface during 1979 to 2018. Only the regions with water column depth in the range of 200-1500 m are shown.
